# Cold stress–induced ferroptosis in liver sinusoidal endothelial cells determines liver transplant injury and outcomes

**DOI:** 10.1172/jci.insight.174354

**Published:** 2024-02-08

**Authors:** Hidenobu Kojima, Hirofumi Hirao, Kentaro Kadono, Takahiro Ito, Siyuan Yao, Taylor Torgerson, Kenneth J. Dery, Hiroaki Kitajima, Takahiro Ogawa, Fady M. Kaldas, Douglas G. Farmer, Jerzy W. Kupiec-Weglinski

**Affiliations:** 1 Dumont-UCLA Transplantation Center, Department of Surgery, Division of Liver and Pancreas Transplantation, David Geffen School of Medicine at UCLA, Los Angeles, California, USA.; 2 Weintraub Center for Reconstructive Biotechnology, Division of Regenerative and Reconstructive Sciences, UCLA School of Dentistry, Los Angeles, California, USA.

**Keywords:** Immunology, Transplantation, Apoptosis pathways

## Abstract

Although cold preservation remains the gold standard in organ transplantation, cold stress–induced cellular injury is a significant problem in clinical orthotopic liver transplantation (OLT). Because a recent study showed that cold stress activates ferroptosis, a form of regulated cell death, we investigated whether and how ferroptosis determines OLT outcomes in mice and humans. Treatment with ferroptosis inhibitor (ferrostatin-1) during cold preservation reduced lipid peroxidation (malondialdehyde; MDA), primarily in liver sinusoidal endothelial cells (LSECs), and alleviated ischemia/reperfusion injury in mouse OLT. Similarly, ferrostatin-1 reduced cell death in cold-stressed LSEC cultures. LSECs deficient in nuclear factor erythroid 2-related factor 2 (NRF2), a critical regulator of ferroptosis, were susceptible to cold stress–induced cell death, concomitant with enhanced endoplasmic reticulum (ER) stress and expression of mitochondrial Ca^2+^ uptake regulator (MICU1). Indeed, supplementing MICU1 inhibitor reduced ER stress, MDA expression, and cell death in NRF2-deficient but not WT LSECs, suggesting NRF2 is a critical regulator of MICU1-mediated ferroptosis. Consistent with murine data, enhanced liver NRF2 expression reduced MDA levels, hepatocellular damage, and incidence of early allograft dysfunction in human OLT recipients. This translational study provides a clinically applicable strategy in which inhibition of ferroptosis during liver cold preservation mitigates OLT injury by protecting LSECs from peritransplant stress via an NRF2-regulatory mechanism.

## Abstract 2

<p>This translational study identifies a clinically-applicable novel strategy to inhibit ferroptosis during organ preservation to mitigate liver transplant injury by protecting LSECs via NRF2 regulation</p>

## Graphical abstract

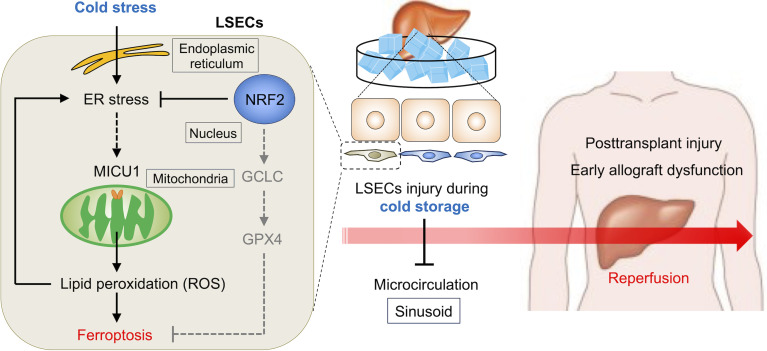

## Introduction

Although orthotopic liver transplantation (OLT) is a treatment of choice for patients with end-stage liver disease and certain hepatic malignancies, donor organ shortages remain a worldwide health problem. Despite the increased use of suboptimal or “marginal” livers from deceased donors, including donations after circulatory death, from the elderly, and with hepatic steatosis greater than 30%, more than 20% of liver grafts were discarded because of poor quality (1). In addition, marginal liver grafts are particularly susceptible to ischemia/reperfusion injury (IRI), an innate immune-driven local inflammatory response, which compromises graft and patient survival and worsens OLT outcomes (1, 2). Therefore, besides surgical techniques, immunosuppressive drug protocols, and intensive care assistance, donor organ preservation is essential for improving clinical outcomes and expanding the donor organ pool available for lifesaving OLT.

Despite recent refinements in liver preservation techniques, including hypothermic oxygenated perfusion, supercooling preservation, and normothermic machine perfusion (NMP) (3–6), static cold storage (SCS) remains the gold standard because of its simplicity and cost-effectiveness (7). In fact, with no significant differences in the incidence of nonanastomotic biliary strictures and graft/patient survival between NMP and SCS liver preservation in early clinical trials (6), NMP can add up to $90,000 to a single OLT procedure (8, 9). However, because of cold stress–induced cellular damage (2, 7), new approaches to reducing cold preservation–driven hepatocellular injury are warranted.

The injury in liver sinusoidal endothelial cells (LSECs) during cold organ preservation represents the initial key factor leading to hepatic IRI, determining poor graft microcirculation, platelet activation, persistent vasoconstriction, oxidative stress response, Kupffer cell activation, and neutrophil infiltration (2, 10). While IRI affects hepatocytes, especially under normothermic stress conditions, LSECs develop profound alterations during cold preservation and are particularly vulnerable to cold stress–induced IRI (10). Despite the apparent clinical importance, the mechanism of cold stress–induced LSEC injury in OLT remains largely unknown.

Ferroptosis, recognized in 2012 as a new nonapoptotic regulated cell death program, occurs due to lethal lipid peroxidation caused by lipid and iron metabolism (11–14). The system x_c_^−^/glutathione (GSH)/glutathione peroxidase 4 (GPX4) pathway is a central inhibitor of lipid peroxidation and ferroptosis (15). However, a recent report has demonstrated that cold stress drives lipid ROS accumulation and ferroptotic cell death platform through a mitochondrial Ca^2+^ uptake regulator, mitochondrial calcium uptake 1 (MICU1) (16). Cold stress also induces endoplasmic reticulum (ER) stress, which causes Ca^2+^ release from the ER into the cytosol (17–19). Thus, ER stress during liver preservation can facilitate mitochondrial Ca^2+^ influx, leading to ferroptosis (20, 21). Despite growing evidence for the contribution of ferroptosis in the pathophysiology of heart, kidney, liver, and intestine IRI (22–26) and the relevance of ferroptosis in cellular injury, no study to our knowledge has investigated the role of cold stress–induced ferroptosis in OLT.

In the current study, we used a well-established murine OLT model with extended cold preservation, mimicking the “marginal” donor livers in clinical settings, and human liver transplants to investigate whether and how ferroptosis can affect OLT. We found that inhibition of ferroptosis during liver cold preservation protected LSECs against IRI following OLT (IRI-OLT) in mice. Indeed, nuclear factor erythroid 2-related factor 2–deficient (NRF2-deficient) (KO) LSECs were particularly susceptible to ER stress and ferroptosis under cold stress. Strikingly, inhibiting MICU1 suppressed ferroptosis in NRF2-deficient (KO) but not NRF2-proficient (WT) LSECs, suggesting NRF2 is critically involved in the MICU1-mediated ferroptosis pathway. In the clinical arm, consistent with the murine findings, increased NRF2 levels in cold-stored human livers reduced lipid peroxidation, mitigated IRI-OLT, and suppressed the incidence of early allograft dysfunction (EAD). This translational study provides a viable clinical strategy in which adjunct blockade of ferroptotic cell death during organ preservation can “rejuvenate” donor livers and improve OLT outcomes by protecting LSECs from cold stress via an NRF2-regulatory mechanism.

## Results

### Prevention of ferroptosis during hepatic cold storage improves mouse OLT.

**Figure 1:**
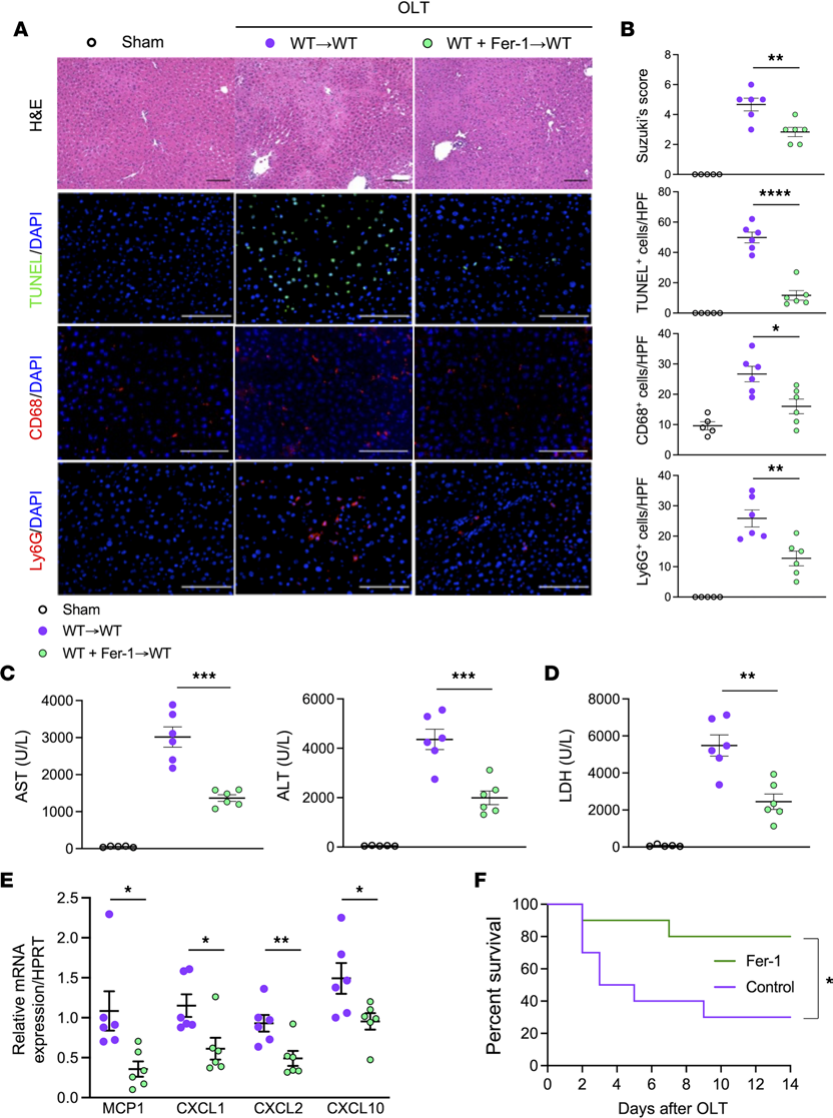
Inhibition of ferroptosis during liver cold storage mitigates the hepatocellular injury and improves mouse OLT survival. Mouse WT livers, stored for 18 hours at 4°C with/without ferroptosis inhibitor (ferrostatin-1; Fer-1, 30 μM) were transplanted into syngeneic WT recipients (*n* = 6/group). Serum and OLT samples were analyzed at 6 hours postreperfusion. The sham group (*n* = 5) underwent the same procedures except for OLT. (**A**) Representative H&E, TUNEL, CD68, and Ly6G staining. Scale bars = 100 μm. (**B**) Suzuki’s histological grading of liver IRI and quantification of TUNEL-, CD68-, and Ly6G-positive cells/high-power field (HPF). (**C**) Serum AST/ALT and (**D**) LDH levels (U/L). (**E**) qRT-PCR–assisted detection of MCP-1, CXCL1, CXCL2, and CXCL10 in OLT (*n* = 6/group). Data were normalized to HPRT gene expression. qRT, quantitative reverse transcription. (**F**) Mouse OLT survival at 14 days (*n* = 10/group) (Kaplan-Meier method). White circle: sham; purple circle: WT OLT; green circle: WT+Fer-1 OLT. Data are shown as mean ± SEM. *P < 0.05, **P < 0.01, ***P < 0.001, ****P < 0.0001, Student’s *t* test (**B**−**E**), log-rank test (**F**).

To assess the role of ferroptosis in mouse IRI-OLT, University of Wisconsin (UW) solution supplemented with ferrostatin-1 (Fer-1), a specific ferroptosis inhibitor, was infused (2 mL) into donor liver grafts before cold storage (18 h/4°C). To optimize Fer-1 concentration, we assessed liver flush for high mobility group box 1 (HMGB1) levels, a danger-associated molecular pattern (DAMP) released during cell death, including ferroptosis (27) (Supplemental Figure 1; supplemental material available online with this article; https://doi.org/10.1172/jci.insight.174354DS1). Cold-stored livers were flushed with lactated Ringer’s solution (2 mL) just before the transplant surgical procedure. Addition of Fer-1 (30 μM) to UW solution during organ preservation significantly mitigated OLT injury (WT→WT), assessed at 6 hours postreperfusion by Suzuki’s histological grading of liver damage (sinusoidal congestion, hepatocellular vacuolization, and necrosis) (28) (mean ± SEM 4.7 ± 0.4 vs. WT+Fer-1→WT = 2.8 ± 0.3, *P* = 0.0056) (Figure 1, A and B), the frequency of TUNEL-positive necrotic/apoptotic cells/high-power field (HPF) (49.8 ± 3.5 vs. WT+Fer-1→WT = 11.7 ± 3.2, *P* < 0.0001) (Figure 1, A and B), and infiltration by CD68-positive macrophages/HPF (26.7 ± 2.6 vs. WT+Fer-1→WT = 16.0 ± 2.4, *P* = 0.0128) and Ly6G-positive neutrophils/HPF (25.8 ± 2.8 vs. WT+Fer-1→WT = 12.7 ± 2.4, *P* = 0.0052) (Figure 1, A and B). In agreement with histological data, plasma levels (U/L) of aspartate aminotransferase (AST), alanine transaminase (ALT), and lactate dehydrogenase (LDH) were significantly lower after supplementing donor livers with Fer-1, as compared with controls (AST = 3,014 ± 274 vs. WT+Fer-1→WT = 1,365 ± 88, *P* = 0.0002; ALT = 4,358 ± 412 vs. WT+Fer-1→WT = 1,992 ± 274, *P* = 0.0007; LDH = 5,485 ± 573 vs. WT+Fer-1→WT = 2,447 ± 416, *P* = 0.0016) (Figure 1, C and D). Thus, blocking ferroptosis during cold preservation of the donor liver alleviated IRI-OLT.

Consistent with Fer-1–mediated reduction of macrophage and neutrophil infiltration in IRI-stressed OLT, treatment with Fer-1 significantly decreased mRNA levels coding for hepatic inflammatory monocyte chemoattractant protein-1 (MCP-1), CXCL1, CXCL2, and CXCL10 (Figure 1E) and improved OLT survival (14-day survival 80% versus 30% in controls, *n* = 10/group, *P* = 0.0258) (Figure 1F). Thus, inhibiting ferroptosis during organ cold storage improved OLT outcomes in mice by mitigating liver IRI and hepatic inflammation.

### Inhibition of ferroptosis during liver preservation protects LSECs against cold stress–induced injury.

**Figure 2:**
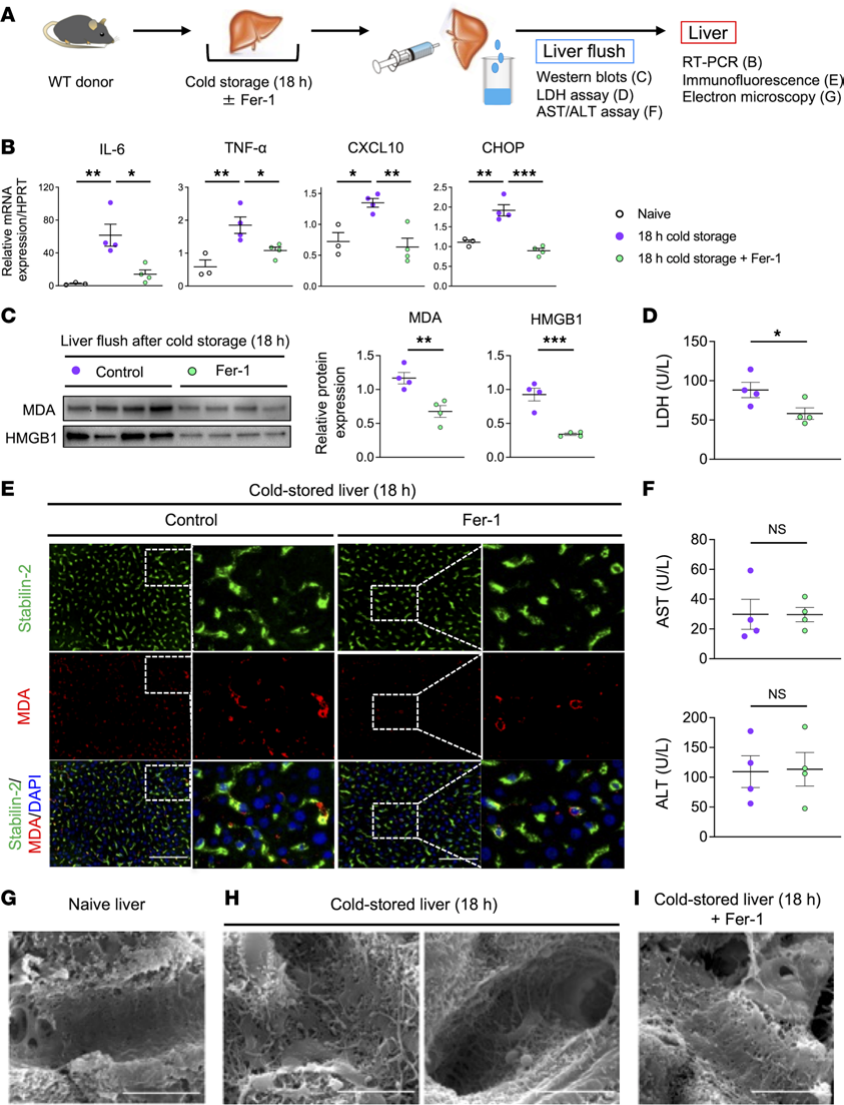
Inhibition of ferroptosis in LSECs reduces the inflammatory signature and MDA levels in cold-stored mouse livers. (**A**) WT livers stored in UW solution (4°C/18 h) with/without ferroptosis inhibitor (Fer-1, 30 μM) were perfused with PBS (2 mL) through a cuff placed at the portal vein to collect liver flush from inferior vena cava. (**B**) qRT-PCR–assisted detection of mRNA coding for IL-6, TNF-α, CXCL10, and CHOP in cold-stored livers (*n* = 3–4/group). Data were normalized to HPRT gene expression. (**C**) Western blot–assisted detection of MDA and HMGB1 levels in the liver flush (5 μL) from cold-stored livers (*n* = 4/group). (**D**) LDH levels (U/L) in the liver flush (*n* = 4/group). (**E**) Representative (*n* = 4/group) immunohistochemical staining of stabilin-2/MDA in cold-stored livers with/without Fer-1. Scale bars = 100 μm. (**F**) AST/ALT levels (U/L) in the liver flush (*n* = 4/group). (**G**–**I**) Scanning electron microscope analysis of naive and cold-stored livers with/without Fer-1. Scale bars = 5 μm. White circle: naive livers; purple circle: cold-stored livers; green circle: cold-stored livers+Fer-1. Data are shown as mean ± SEM. *P < 0.05, **P < 0.01, ***P < 0.001, 1-way ANOVA followed by Tukey’s honestly significance difference (HSD) test (**B**), Student’s *t* test (**C**, **D**, and **F**). CHOP, CCAAT-enhancer-binding protein homologous protein; MDA, malondialdehyde.

We next evaluated cold-stored mouse livers with/without Fer-1 treatment (Figure 2A). Ferroptosis blockade significantly decreased mRNA levels coding for proinflammatory IL-6, TNF-α, and CXCL10 as compared with controls (Figure 2B). It mitigated otherwise-elevated CHOP induced by ER stress (Figure 2B). These results document that inhibition of ferroptosis in the donor liver attenuated the inflammatory signature and ER stress response in cold-stored OLT.

Malondialdehyde (MDA), an end product of ROS-mediated lipid peroxidation, is essential for ferroptosis (15, 29), while serum MDA levels released from stressed cells of the damaged tissue are associated with organ dysfunction (30, 31). As expected, Fer-1 adjunct added during organ cold storage significantly reduced MDA and HMGB1 release into the liver flush (Figure 2C). Because LDH release, a measure of plasma membrane damage, significantly decreased after Fer-1 treatment (Figure 2D), we propose that ferroptosis blockade alleviated lipid peroxidation and cell death in liver grafts.

To find out which liver cells suffered from ferroptosis during organ cold preservation, we used immunofluorescence double staining for MDA and albumin (hepatocytes), stabilin-2 (LSECs), or C-type lectin domain family 4, member F (Clec4F) (Kupffer cells), which are major phenotypic markers of parenchymal (hepatocytes) and nonparenchymal (LSECs and Kupffer cells) liver cells. Interestingly, most of MDA overlapped with stabilin-2 but not albumin or Clec4F expression (Figure 2E and Supplemental Figure 2, A and B), suggesting that LSECs were the primary source of lipid peroxidation. Comparable AST/ALT levels in the liver flush after treatment with Fer-1 (Figure 2F) verified that ferroptosis blockade failed to affect the hepatocellular function in cold-stressed livers.

Next, we used scanning electron microscopy to evaluate the morphology of LSECs. Naive livers were characterized by fenestrations clustered on LSECs (Figure 2G). In contrast, shrunken LSECs with lost fenestration and bare sinusoids without LSECs were readily detected in cold-stored livers (Figure 2H), validating severe LSEC injury. However, the morphology of LSECs and sinusoids was well preserved after treatment of donor liver with Fer-1 during the 18-hour period of cold preservation in UW solution (Figure 2I). Collectively, inhibition of ferroptosis reduced MDA and DAMP release while protecting LSECs from cold storage–inflicted hepatic injury. Since LSEC damage may result in platelet aggregation and deteriorate liver microcirculation (10), we verified Fer-1 treatment effectively reduced platelet aggregation, as measured by integrin αIIb expression in postreperfusion murine livers (Supplemental Figure 3, A and B).

### Cold stress enhances MICU1, lipid peroxidation, and NRF2 expression in LSECs.

**Figure 3:**
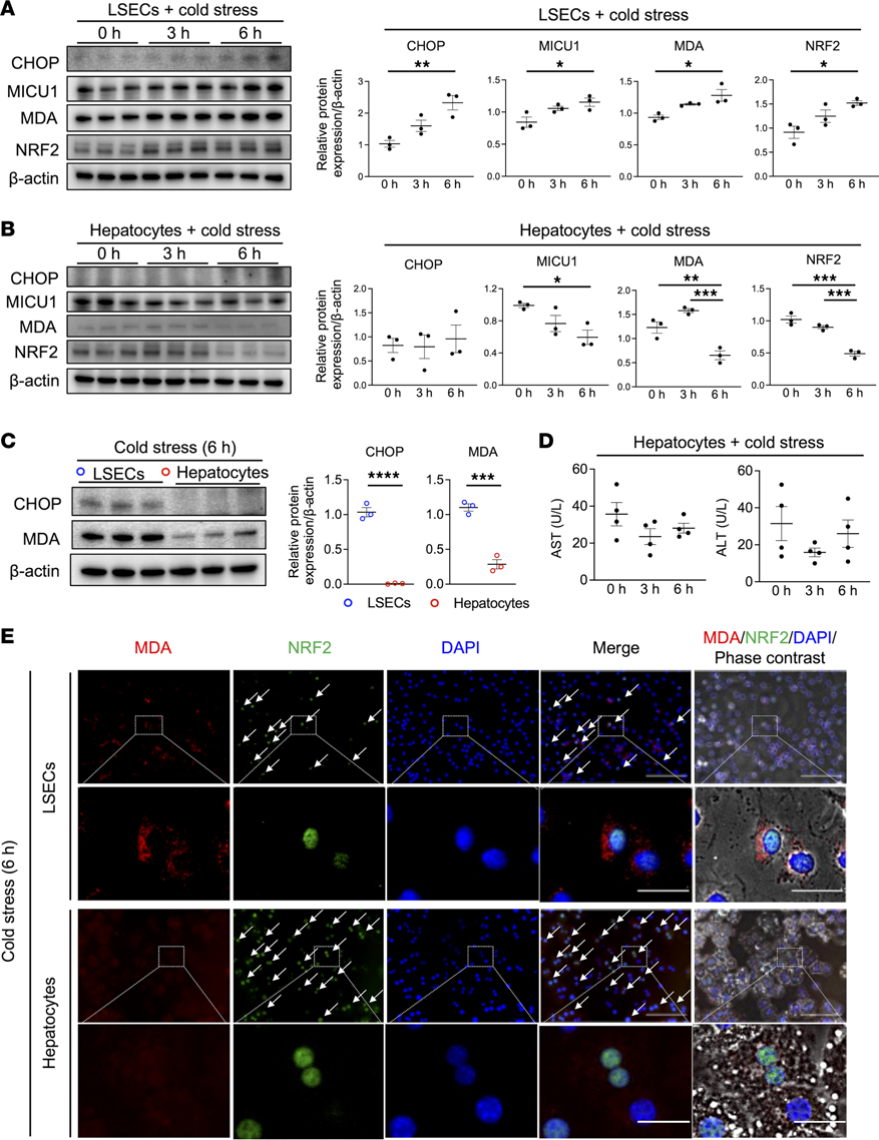
Cold stress enhances ER stress, MICU1, MDA, and NRF2 expression in LSEC cultures. (**A**) Primary WT LSEC and (**B**) hepatocyte cultures were subjected to cold stress (4°C) for 0–6 hours. Western blot–assisted detection and relative intensity ratio of CHOP, MICU1, MDA, and NRF2. Expression of β-actin served as the internal control and was used for normalization (*n* = 3/group). (**C**) Western blot–assisted detection and relative intensity ratio of CHOP and MDA in LSEC and hepatocyte cultures. Expression of β-actin served as the internal control and was used for normalization (*n* = 3/group). Blue circle: LSECs, red circle: hepatocytes. (**D**) AST/ALT levels (U/L) in culture medium of hepatocyte cultures (*n* = 4/group). (**E**) Representative immunohistochemical staining of MDA/NRF2 in LSEC and hepatocyte cultures (*n* = 3/group). Arrows indicate nuclear localization of NRF2. Scale bars = 100 μm in low and 20 μm in high magnification. Data are shown as mean ± SEM. *P < 0.05, **P < 0.01, ***P < 0.001, ****P < 0.001, 1-way ANOVA followed by Tukey’s HSD test (**A**, **B**, and **D**), Student’s *t* test (**C**).

Having demonstrated that LSECs, rather than hepatocytes, were susceptible to ferroptosis in vivo, we focused on underlying mechanisms utilizing well-controlled cell culture systems. MICU1, a gatekeeper in mitochondrial Ca^2+^ uptake, is required for lipid peroxidation and ferroptosis during cold stress (16), and we found that unlike in hepatocytes, MICU1/MDA was upregulated in cold-stressed LSECs in vitro (Figure 3, A and B). As ER stress leads to cytosolic Ca^2+^ increase (17–19), it can also promote ferroptosis via mitochondrial Ca^2+^ influx. Similar to an MICU1/MDA profile, we found increased CHOP expression selectively in LSECs (Figure 3, A and B). Collectively, ER stress, MICU1, and lipid peroxidation were induced in cold-stressed LSECs but not in hepatocyte cultures.

We further evaluated the role of a key molecular ferroptosis regulator, GSH/GPX4, which is under the control of the NRF2/glutamate-cysteine ligase catalytic subunit (GCLC) axis (13, 32). Contrary to our expectations, cold stress itself failed to enhance GCLC/GPX4 expression in LSEC and hepatocyte cultures (Supplemental Figure 4, A and B). Having shown that NRF2 expression was upregulated selectively in LSECs (Figure 3, A and B), we assumed that, unlike GPX4, NRF2 signaling was essential in the mechanism of cold stress–induced LSEC injury. Of note, we observed downregulated cleaved caspase-3 (a necessary step for apoptosis) and unchanged receptor interacting protein 3 (RIP3) (a necroptosis marker) levels that are consistent with the idea that neither apoptosis nor necroptosis played a role in cold stress–triggered LSEC damage (Supplemental Figure 4, C and D).

To focus on cell susceptibility to cold stress, we found that CHOP/MDA expression was significantly higher in LSECs than in hepatocytes following cold stress (Figure 3C), while similar AST/ALT levels in hepatocyte culture media implied little effect on the hepatocyte integrity (Figure 3D). NRF2 translocates to the nucleus as a transcriptional activator for antioxidant response (33). Although immunofluorescence showed nuclear localization of NRF2 in LSECs and hepatocytes, MDA-expressing cells were more abundant in damaged LSECs (Figure 3E). Thus, although NRF2 signaling was protective against oxidative stress in LSECs and hepatocytes, LSECs appeared more vulnerable to lipid peroxidation.

### NRF2 signaling suppresses cold stress–induced ER stress, MICU1, lipid peroxidation, and ferroptosis in LSECs.

**Figure 4:**
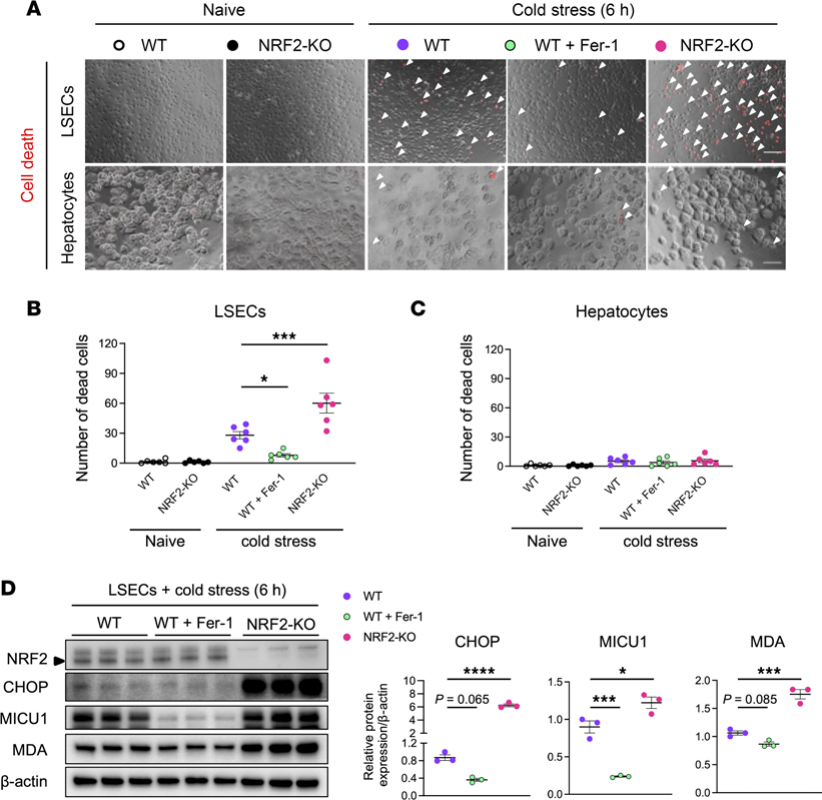
NRF2 deficiency exacerbates cold stress–induced ferroptotic cell death and enhances ER stress, MICU1, and MDA expression in LSEC cultures. (**A**) Representative (*n* = 6/group) images of dead cell detection in WT versus NRF2-deficient (NRF2-KO) LSEC and hepatocyte cultures with/without cold stress (4°C). Arrowheads indicate dead cells. Scale bars = 100 μm. Fer1, ferrostatin-1 (10 μM). Quantification of dead cells in (**B**) LSEC and (**C**) hepatocyte cultures (*n* = 6/group). (**D**) Western blot–assisted detection and relative intensity ratio of NRF2, CHOP, MICU1, and MDA in LSEC cultures. Expression of β-actin served as the internal control and was used for normalization (*n* = 3/group). White circle: naive WT; black circle: naive NRF2-KO; purple circle: WT+cold stress; green circle: WT+cold stress+Fer-1; pink circle: NRF2-KO+cold stress. Data are shown as mean ± SEM. *P < 0.05, ***P < 0.001, ****P < 0.001, 1-way ANOVA followed by Dunnett’s multiple comparisons test (**B**−**D**).

To assess whether NRF2 signaling regulates ferroptosis, we compared LSEC and hepatocyte cultures from NRF2-proficient (WT) and NRF2-deficient (KO) mice. We verified that adjunct Fer-1 significantly reduced cell death in WT and NRF2-KO LSECs (Figure 4, A and B, and Supplemental Figure 5A). However, unlike hepatocytes, NRF2-deficient LSECs were susceptible to cold stress–induced cell death compared with WT counterparts (Figure 4, A–C), suggesting NRF2 signaling regulated cell death selectively in LSECs.

As CHOP, MICU1, and MDA expression was significantly higher in NRF2-KO compared with WT LSECs, NRF2 deficiency augmented ER stress and lipid peroxidation, concomitant with enhanced MICU1 expression in response to the cold stress (Figure 4D). Notably, Fer-1 treatment reduced MICU1, CHOP, and MDA levels in WT LSECs, suggesting inhibition of ferroptosis impacted MICU1 degradation in addition to ER stress and lipid peroxidation (Figure 4D). Like in the cell death assay, neither Fer-1 treatment nor NRF2 signaling influenced CHOP/MDA expression in cold-stressed hepatocytes (Supplemental Figure 5B). Taken together, unlike in hepatocytes, NRF2 signaling reduced ER stress, MICU1 expression, lipid peroxidation, and ferroptotic cell death in cold-stressed LSECs.

### NRF2-mediated signaling pathway protects liver grafts against cold stress–induced injury.

**Figure 5:**
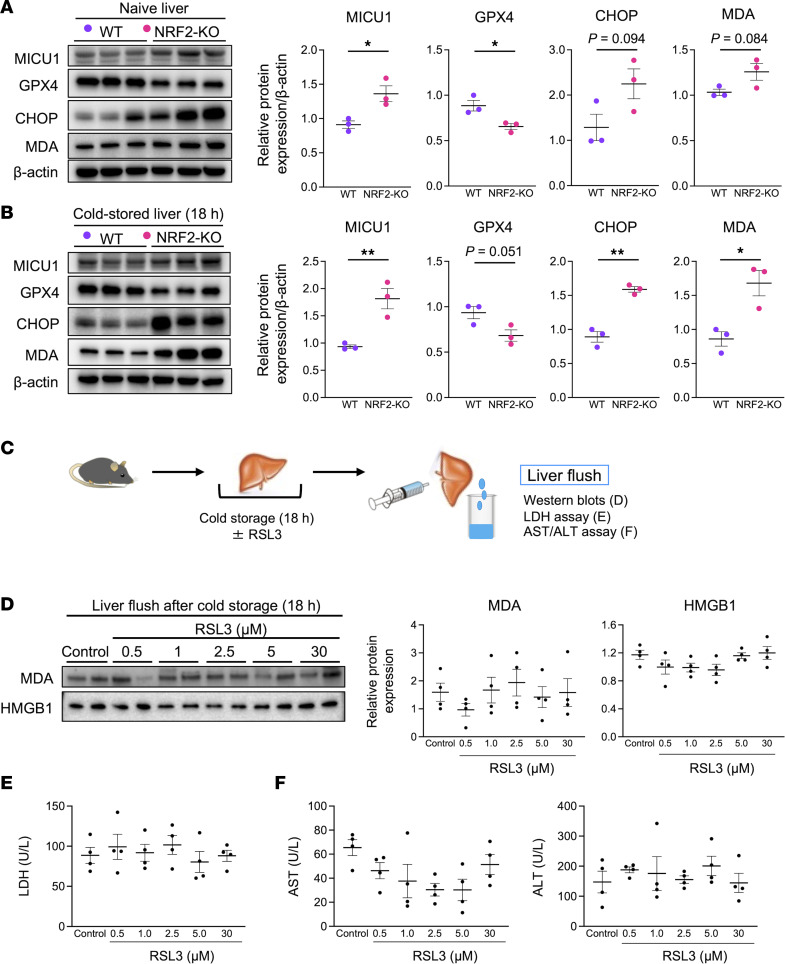
GPX4 pathway is dispensable in cold stress–induced liver injury. Western blot–assisted detection and relative intensity ratio of MICU1, GPX4, CHOP, and MDA in WT and NRF2-deficient (NRF2-KO) (**A**) naive livers and (**B**) 18-hour cold-stored livers. Expression of β-actin served as the internal control and was used for normalization (*n* = 3/group). (**C**) WT livers stored in UW solution (4°C/18 h) with/without RSL3 (GPX4 inhibitor) were perfused with PBS (2 mL) through a cuff placed at the portal vein to collect liver flush from inferior vena cava. (**D**) Western blot–assisted detection of MDA and HMGB1 in the liver flush (5 μL) from cold-stored livers (*n* = 4/group). (**E**) LDH and (**F**) AST/ALT levels (U/L) in the liver flush (*n* = 4/group). Purple circle: WT livers; pink circle: NRF2-KO livers. Data are shown as mean ± SEM. *P < 0.05, **P < 0.01, Student’s *t* test (**A** and **B**), 1-way ANOVA followed by Tukey’s HSD test (**D**−**F**).

To assess whether NRF2-mediated signaling regulates ferroptosis in liver grafts, we analyzed the expression of MICU1 and GPX4, in addition to ER stress and lipid peroxidation, in WT and NRF2-KO livers with/without cold stress. Increased MICU1 and decreased GPX4 expression were seen with disruption of NRF2 signaling in both naive and cold-stored groups (Figure 5, A and B). Notably, while CHOP and MDA trended lower in naive WT compared with NRF2-KO liver grafts, a significant increase of both was noted in cold-stressed NRF2-deficient livers (CHOP: naive *P* = 0.094 and cold-stored liver *P* = 0.0014; MDA: naive *P* = 0.084 and cold-stored liver *P* = 0.0186) (Figure 5, A and B). These data indicate that NRF2 signaling was essential to suppress cold stress–induced ER stress and lipid peroxidation. We verified that the primary source of enhanced MDA expression in NRF2-KO livers derived from LSECs by increased stabilin-2 staining (Supplemental Figure 6).

Although NRF2 signaling enhanced GPX4 expression in liver grafts (Figure 5, A and B), the GPX4 pathway might not be essential in the mechanism of cold stress–induced ferroptosis (Supplemental Figure 4, A and B). To address this potentially conflicting finding, we supplied UW solution with a specific GPX4 inhibitor (RSL3; 0.5–30 μM) during liver cold preservation (Figure 5C). As we observed no difference in MDA, HMGB1, LDH, AST, and ALT release into the liver flush between RSL3-treated samples versus controls (Figure 5, D−F), we conclude that NRF2, but not GPX4 signaling, regulated ferroptosis in our model.

### MICU1 mediates cold stress–induced ferroptosis in LSECs in an NRF2-dependent manner.

**Figure 6:**
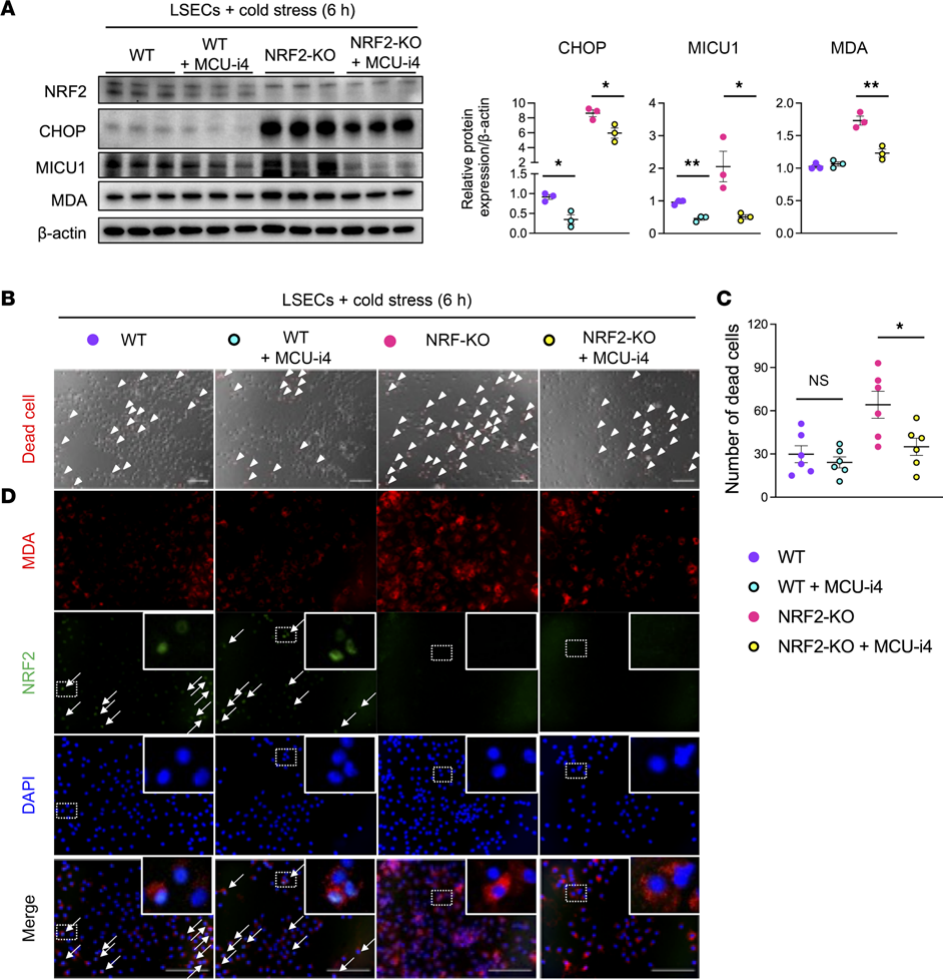
MICU1 inhibition reduces cold stress–induced ferroptotic cell death and MDA expression in NRF2-deficient LSECs. (**A**) Western blot–assisted detection and relative intensity ratio of NRF2, CHOP, MICU1, and MDA in cold-stressed LSECs (6 h/4°C). Expression of β-actin served as the internal control and was used for normalization (*n* = 3/group). (**B**) Representative (*n* = 6/group) images of dead cell detection in WT and NRF2-KO LSECs with/without MICU1 inhibitor (MCU-i4; 10 μM). Arrowheads indicate dead cells. Scale bars = 100 μm. (**C**) Quantification of dead cells (*n* = 6/group). (**D**) Representative (*n* = 3/group) immunohistochemical staining of MDA/NRF2 in WT and NRF2-KO LSECs with/without MICU1 inhibitor (MCU-i4). Scale bars = 100 μm. Arrows indicate nuclear localization of NRF2. Purple circle: WT; green circle: WT+MCU-i4; pink circle: NRF2-KO; yellow circle: NRF2-KO+MCU-i4. Data are shown as mean ± SEM. *P < 0.05, **P < 0.01, Student’s *t* test (**A** and **D**).

To study whether NRF2 signaling may affect the regulatory function of MICU1 in the mechanism of ferroptosis, we incubated WT and NRF2-deficient LSECs with MICU1 inhibitor (MCU-i4; 10 μM) and evaluated ER stress/lipid peroxidation in cell cultures. Interestingly, while adding MCU-i4 depressed CHOP expression in WT and NRF2-deficient LSECs, MICU inhibitor selectively reduced MDA in NRF2-KO but not WT LSEC cultures (Figure 6A). Consistent with lipid peroxidation, MCU-i4 significantly reduced cell death in NRF2-deficient but not WT LSECs (Figure 6, B and C). Collectively, the MICU1 inhibitor suppressed ER stress, lipid peroxidation, and cell death in NRF2-deficient LSECs, suggesting that NRF2 might be a critical regulator in the MICU1-mediated ferroptosis pathway. Immunofluorescence staining of MDA/NRF2 validated nuclear localization of NRF2 in WT LSECs and enhanced MDA expression in untreated NRF2-KO LSECs (Figure 6D), Of note, MCU-i4 adjunct significantly reduced MICU1 expression in WT and NRF2-KO stressed LSECs (Figure 6A). This finding is consistent with the previously demonstrated ability of Fer-1 to attenuate MICU1 expression in cold-stressed LSECs (Figure 4D).

### MICU1 antagonism during organ cold storage attenuates MDA/HMGB1 release and OLT injury in NRF2-deficient OLT.

**Figure 7:**
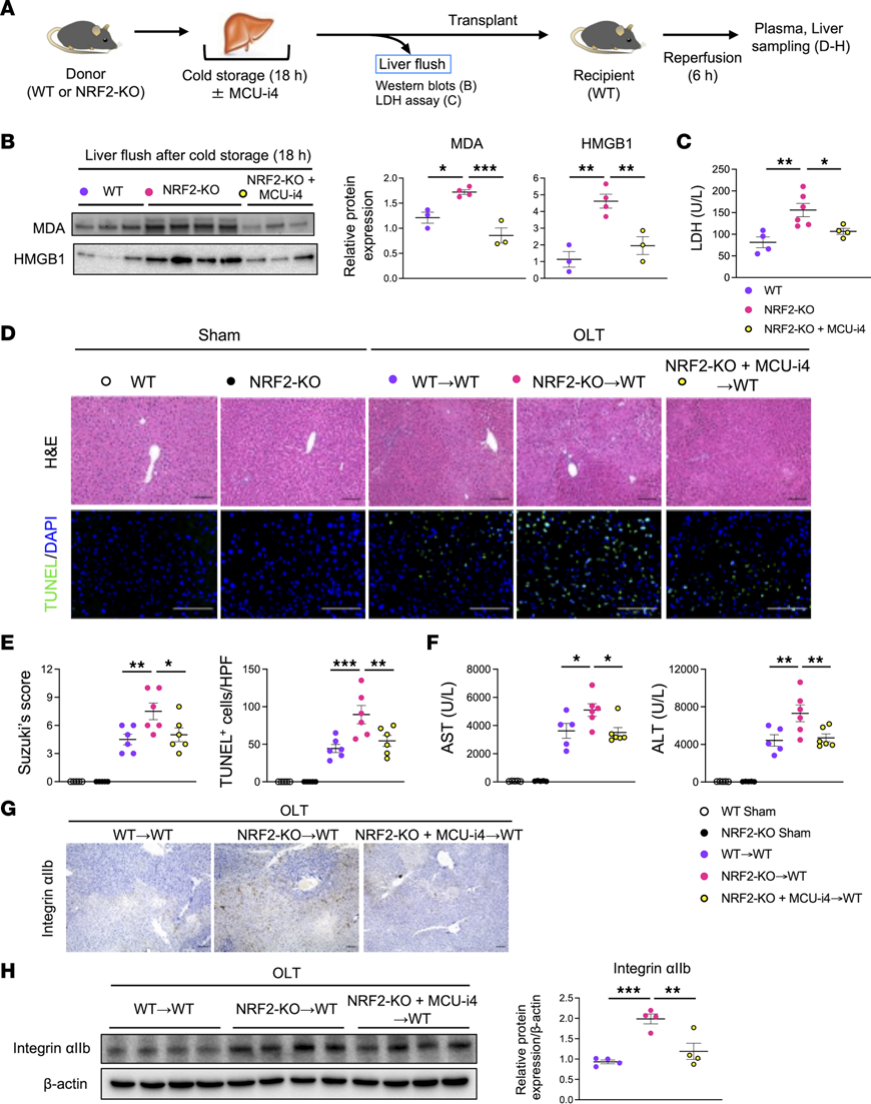
Adjunct MICU1 inhibition during cold storage alleviates the hepatocellular injury in NRF2-KO liver grafts. (**A**) WT and NRF2-KO livers stored in UW solution (4°C/18 h) with/without MICU1 inhibitor (MCU-i4; 10 μM) were perfused with PBS (2 mL) through a cuff placed at the portal vein to collect liver flush from inferior vena cava. They were then transplanted into WT recipients (*n* = 5−6/group). Serum and OLT samples were analyzed at 6 hours after reperfusion. The sham group underwent the same procedures except for OLT. (**B**) Western blot–assisted detection of MDA and HMGB1 levels in the liver flush (5 μL) from cold-stored livers (*n* = 3−4/group). (**C**) LDH levels (U/L) in the liver flush (*n* = 4−6/group). (**D**) Representative (*n* = 5−6/group) H&E and TUNEL staining. Scale bars = 100 μm. (**E**) Suzuki’s histological grading of liver IRI and quantification of TUNEL-positive cells/HPF in OLT. (**F**) Serum AST and ALT levels (U/L). (**G**) Representative (*n* = 5−6/group) integrin αIIb staining. Scale bars = 100 μm. (**H**) Western blot–assisted detection and relative intensity ratio of integrin αIIb in OLT. Expression of β-actin served as the internal control and was used for normalization (*n* = 4/group). White circle: WT sham; black circle: NRF2-KO sham; purple circle: WT OLT; pink circle: NRF2-KO OLT; yellow circle: NRF2-KO+MCU-i4 OLT. Data are shown as mean ± SEM. *P < 0.05, **P < 0.01, ***P < 0.001, 1-way ANOVA followed by Dunnett’s multiple comparisons test (**B**, **C**, **E**, **F**, and **H**).

To verify in vivo relevance of our in vitro findings, we supplemented UW solution with MCU-i4 while preserving NRF2-KO livers (Figure 7A). Consistently, significantly increased MDA, HMGB1, and LDH release into the liver flush of NRF2-KO livers was diminished after MCU-i4 treatment (Figure 7, B and C), suggesting that MICU antagonism reduced lipid peroxidation and DAMPs’ release in NRF2-deficient livers.

Last, we implanted NRF2-KO livers with/without MCU-i4 pretreatment into WT recipients and evaluated OLT injury at 6 hours after reperfusion (Figure 7, D−F). Indeed, disruption of NRF2 signaling augmented IRI-OLT, evidenced by Suzuki’s histological score (WT→WT = 4.5 ± 0.6 vs. NRF2-KO→WT = 7.5 ± 0.9, *P* = 0.0060), the frequency of TUNEL-positive cells/HPF (WT→WT = 44.3 ± 5.4 vs. NRF2-KO→WT = 89.5 ± 12.2, *P* = 0.0006), and AST/ALT levels (AST: WT→WT = 3,621 ± 526 vs. NRF2-KO→WT = 5,103 ± 446, *P* = 0.0255; ALT: WT→WT = 4,422 ± 610 vs. NRF2-KO→WT = 7,308 ± 895, *P* = 0.0047). However, adjunct MCU-i4 during organ preservation mitigated OLT damage in NRF2-KO grafts (Suzuki’s histological score: NRF2-KO→WT = 7.5 ± 0.9 vs. NRF2-KO+ MCU-i4 →WT = 5.0 ± 0.7, *P* = 0.0238; the frequency of TUNEL-positive cells/HPF: NRF2-KO→WT = 89.5 ± 12.2 vs. NRF2-KO+MCU-i4→WT = 54.5 ± 7.3, *P* = 0.0071; AST: NRF2-KO→WT = 5,103 ± 446 vs. NRF2-KO+ MCU-i4→WT = 3,508 ± 348, *P* = 0.0108; ALT: NRF2-KO→WT = 7,308 ± 895 vs. NRF2-KO+MCU-i4→WT = 4,692 ± 421, *P* = 0.0072).

Since LSEC injury leads to platelet activation (10), we evaluated platelet aggregation in WT and NRF2-KO liver grafts by immunohistochemistry. Indeed, staining for integrin αIIb revealed augmented platelet deposition in NRF2-KO compared with WT OLT (Figure 7G). However, MCU-i4 treatment reduced platelet aggregation in NRF2-deficient OLT (Figure 7G), indicating LSECs’ protection against stress-induced injury. Western blots verified enhanced integrin αIIb protein levels in NRF2-KO OLT, compared with WT OLT, were attenuated after adjunctive use of MCU-i4 during liver cold storage (Figure 7H).

### Hepatic NRF2 reduces lipid peroxidation and HMGB1 release in discarded human liver grafts.

**Figure 8:**
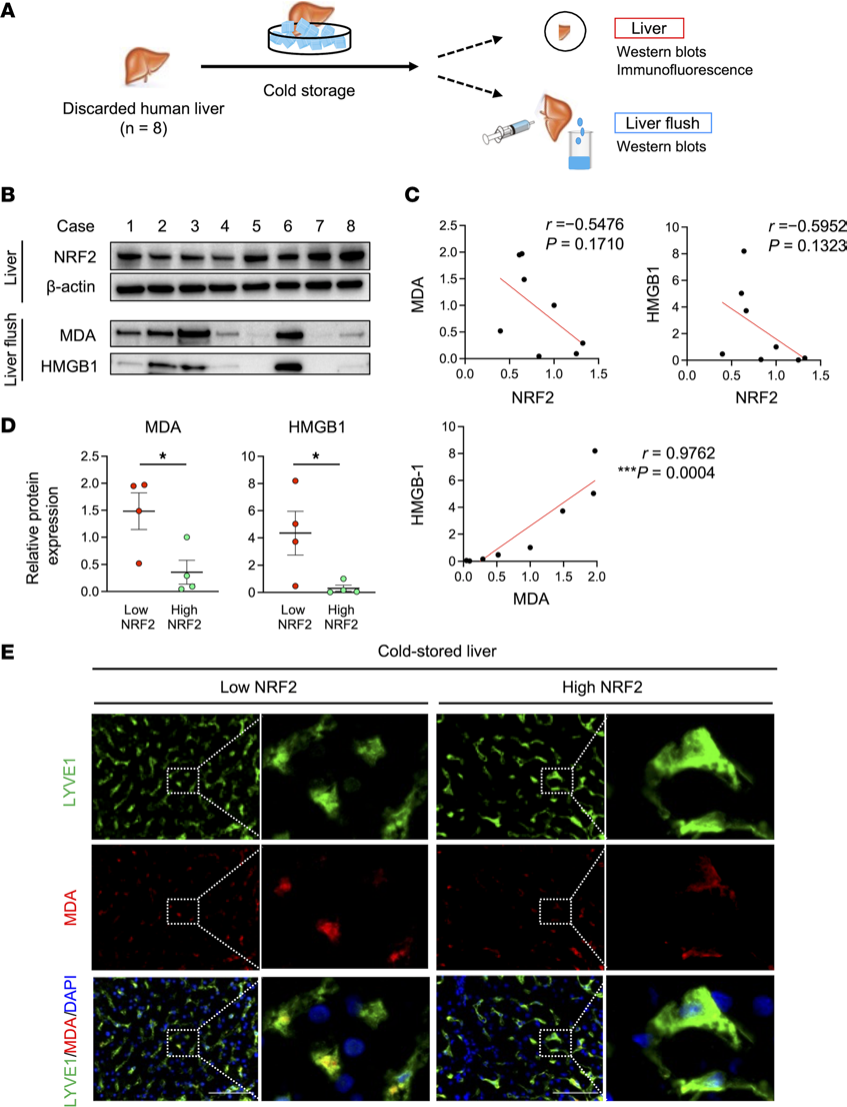
NRF2 signaling suppresses MDA and HMGB1 release into the liver flush in discarded human livers. (**A**) Discarded human liver grafts (*n* = 8) stored in UW solution (4°C) were perfused with UW solution (4 L) through a catheter inserted into the portal vein. Liver biopsies and flush samples were analyzed with Western blots and immunostaining. (**B**) Western blot–assisted detection of NRF2 expression in the liver and MDA/HMGB1 levels in the flush (5 μL). Liver expression of β-actin served as an internal control and was used for normalization. (**C**) Relationship between NRF2 in the liver and MDA in the liver flush, NRF2 in the liver and HMGB1 in the liver flush, and MDA and HMGB1 in the liver flush. (**D**) MDA and HMGB1 levels in low- versus high-NRF2 livers (*n* = 4/group). (**E**) Representative immunohistochemical staining of LYVE1/MDA in cold-stored discarded human livers. Scale bars = 100 μm. Data are shown as mean ± SEM. *P < 0.05, ***P < 0.001, nonparametric Spearman’s method (**C**), Student’s *t* test (**D**).

Having shown NRF2 signaling reduced lipid peroxidation and DAMPs’ release during liver cold storage in mice, we next aimed to validate clinical relevance by collecting liver flush from 8 discarded human livers (OneLegacy Procurement Organization), which were refused because of poor quality (Figure 8A). The demographic donor data and organ information are shown (Supplemental Table 1). Figure 8B illustrates Western blot–assisted detection of NRF2 in discarded human livers and MDA/HMGB1 levels in the liver flush. We detected a negative, albeit insignificant, correlation between NRF2 expression in the liver and MDA/HMGB1 levels in the liver flush (*r* = −0.5476 and −0.5952, respectively) (Figure 8C). Interestingly, however, there was a highly significant positive correlation between MDA and HMGB1 in the liver flush (*r* = 0.9763, *P* = 0.0004) (Figure 8C). Next, after dividing human liver discards, based on Western blot–assisted NRF2 expression, we found MDA and HMGB1 levels in the liver flush were significantly higher in the low compared with the high NRF2 expression group (Figure 8D), suggesting NRF2 suppressed lipid peroxidation and DAMPs’ release in cold-stored livers. Consistent with murine findings, immunofluorescence staining revealed LSECs (LYVE1^+^) were the primary source of lipid peroxidation (MDA^+^) in both NRF2-high- and NRF2-low-expressing discarded human livers (Figure 8E).

### Donor hepatic NRF2 reduces hepatocellular damage and the incidence of EAD in human OLT recipients.

**Figure 9:**
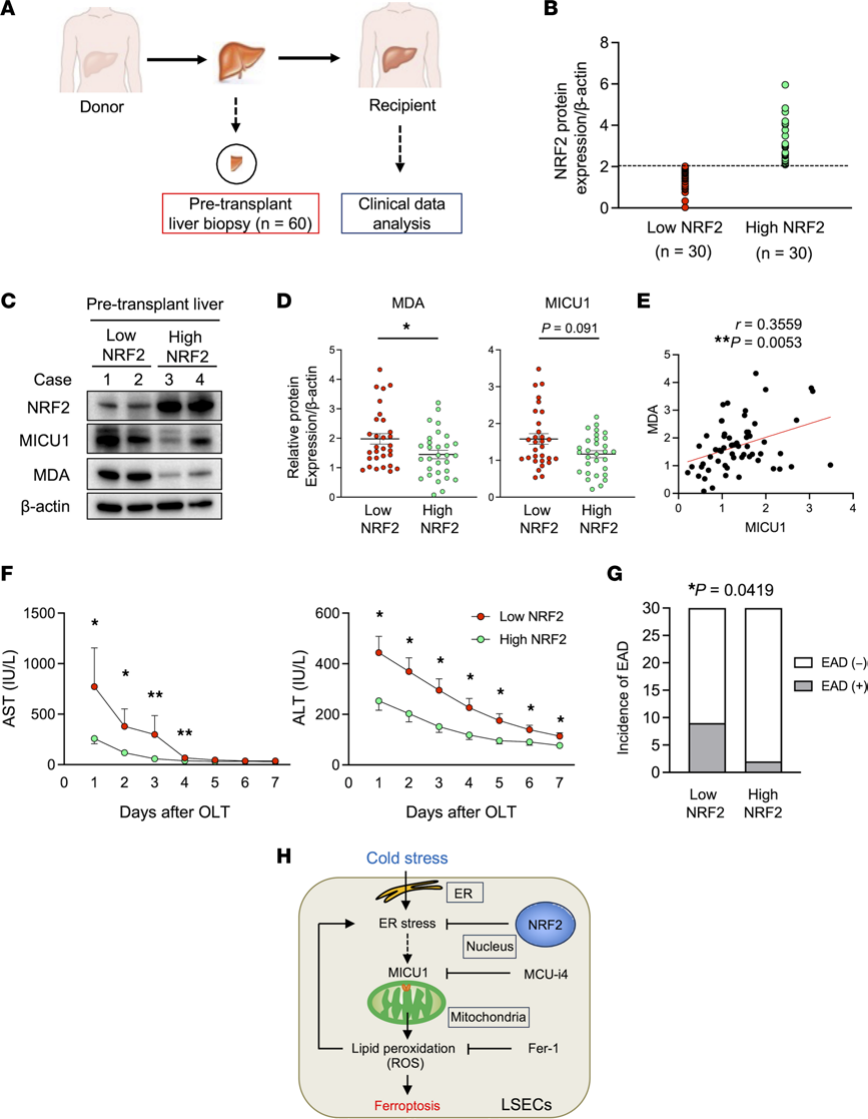
Liver NRF2 suppresses MDA expression and reduces the hepatocellular damage in human OLT. (**A**) Human liver biopsies obtained after cold storage (*n* = 60) were assessed by Western blots. (**B**) Liver grafts were divided into low (*n* = 30) versus high (*n* = 30) NRF2 expression groups, based on the median value of relative NRF2/β-actin levels (cutoff = 2.05). (**C**) Representative Western blots of hepatic NRF2, MICU1, and MDA (case 1/2: low NRF2, case 3/4: high NRF2 levels). (**D**) Western blot–assisted MDA and MICU1 expression. Expression of β-actin served as the internal control and was used for normalization (*n* = 30/group). (**E**) Relationship between hepatic MDA and MICU1 expression. (**F**) Serum AST and ALT levels (U/L) in OLT recipients at POD 1−7. (**G**) EAD incidence in OLT recipients. (**H**) Mechanistic scheme of cold stress–induced ferroptosis cell death pathway in LSECs. Data are shown as mean ± SEM. *P < 0.05, **P < 0.01, Mann-Whitney *U* test (**D** and **F**), nonparametric Spearman’s method (**E**), Fisher’s exact test (**G**).

To investigate the association between NRF2 levels in donor livers and OLT outcomes in human patients, we analyzed 60 clinical liver biopsies collected at the back table before implantation (Figure 9A). These were classified into low- and high-NRF2 groups (*n* = 30/group), based on the median value of relative NRF2/β-actin protein levels (Figure 9B). Besides total bilirubin levels trending higher in the low- versus high-NRF2 group (median = 0.8, range = 0.3–4.9 vs. median = 0.6, range = 0.2–2.9; *P* = 0.054), the patients’ demographic data/clinical parameters did not reveal significant differences (Supplemental Table 2, A and B). Representative Western blots of NRF2, MICU1, and MDA (cases 1 and 2 — low NRF2, cases 3 and 4 — high NRF2) are shown (Figure 9C). Although the high-NRF2 group had significantly lower liver MDA levels (Figure 9D), there was a trend, albeit not significant, of lower MICU1 expression in the high- versus low-NRF2 group (Figure 9D). Notably, MICU1 significantly correlated with the MDA expression in human donor livers (*r* = 0.3559, *P* = 0.0053) (Figure 9E).

We then analyzed clinical OLT outcomes in low- and high-NRF2 groups. The high-NRF2 patients showed decreased OLT damage, exhibiting significantly lower levels of AST (postoperative days [POD] 1–4) and ALT (POD 1–7) (Figure 9F). Strikingly, EAD incidence was significantly lower in the high compared with the low NRF2 expression patient cohort (Figure 9G). Thus, NRF2 signaling in human donor livers reduced lipid peroxidation and improved OLT outcomes.

## Discussion

Our current study documented the contribution of the ferroptotic programmed cell death pathway during donor liver cold storage to liver transplantation outcomes in mice and humans. We used a clinically relevant OLT murine model with extended cold storage in the experimental arm, to clarify whether and how ferroptosis affects cold stress–induced hepatocellular damage. Indeed, inhibition of ferroptosis selectively protected LSECs from cold stress–induced injury, leading to improved hepatic microcirculation and mitigation of postreperfusion early OLT damage. We found that cold stress enhanced ER stress response and lipid peroxidation in LSECs, but not hepatocytes, and that NRF2 regulated MICU1-mediated ferroptosis. Interestingly, GPX4, a central ferroptosis regulator, was dispensable in the mechanism of cold stress–induced liver graft ferroptosis. In the clinical arm, we first verified that LSECs were the prime source of lipid peroxidation in discarded human livers. Then, consistent with murine findings, we found that NRF2 signaling in the donor organ reduced cold stress–induced lipid peroxidation, liver IRI, and the incidence of EAD in human OLT recipients. This translational study provides the first evidence to our knowledge for the role of ferroptosis cell death pathway in “rejuvenating” cold-stored livers in OLT recipients.

Cold storage decreases energy and oxygen demand to minimize ischemic organ damage. As a cost-efficient and convenient method for organ preservation, SCS has been the gold standard for donor organ storage since the 1960s (7). Previous studies suggested that apoptosis occurs only after reperfusion of transplanted organs and that cold storage alone does not trigger apoptosis (34–36). Indeed, we reported that the frequency of TUNEL-positive cells did not increase in cold-stored liver grafts (18 h/4°C) (37). Consistently, our current study showed neither cleaved caspase-3 (a necessary step for apoptosis) nor RIP3 (a necroptosis marker) were upregulated in cold-stressed LSECs or hepatocyte cultures (Supplemental Figure 4, C and D). Nevertheless, as cold stress induces cellular injury, especially in endothelial cells, resulting in poor hepatic microcirculation and liver IRI cascade (2, 10, 38), other signaling pathways besides apoptosis or necroptosis must be essential in the mechanism of endothelial cell damage. Although a transcription factor, Kruppel-like factor 2, which regulates endothelial vasoprotective phenotype, has been reported to decrease in LSECs during organ cold storage due to the lack of shear stress, leading to the deterioration of liver microcirculation (39–41), the underlying mechanisms of LSEC injury in OLT remain largely unknown.

MICU1, a gatekeeper for mitochondrial Ca^2+^ uptake, plays a dual role, in which it preserves mitochondrial Ca^2+^ concentration under basal conditions on one hand while activating mitochondrial Ca^2+^ uptake following ER calcium release (42, 43). Cold stress can elicit ER stress and enhance Ca^2+^ release in the liver, leading to mitochondrial Ca^2+^ overload (17, 19, 44, 45). These reports are linked with a recent study documenting that mitochondrial Ca^2+^ influx through MICU1 facilitates cold stress–induced lipid ROS accumulation and consequent ferroptosis cell death pathway (16). Since the ER regulates intracellular calcium homeostasis, cold-stressed ER can be the source of mitochondrial Ca^2+^ influx, leading to ferroptosis (20, 21). Interestingly, our data showed that treatment with Fer-1 or MCU-i4 suppressed CHOP and MICU1 expression in cold-stressed LSECs (Figure 4D and Figure 6A), indicating that inhibition of ferroptosis might attenuate ER stress and MICU1 signaling. It was reported that mitochondrial generation of ROS cycles back to the ER, leading to further Ca^2+^ release (45). Therefore, increased ER calcium release in the presence of ROS can enhance MICU1, a mitochondrial Ca^2+^ gatekeeper. Collectively, our data are consistent with the idea that suppression of cold stress–induced mitochondria ROS by Fer-1 or MCU-i4 can attenuate ER stress, resulting in MICU1 suppression (Figure 9H). Thus, inhibiting ferroptosis in LSECs during liver cold storage may promote cytoprotection via a feedback mechanism. Future studies need to reveal putative crosstalk between ER stress, MICU1, and ferroptosis platforms under cold stress conditions.

Treatment with MICU1 inhibitor (MCU-i4) reduced ferroptosis in NRF2-deficient but not NRF2-proficient (WT) LSECs (Figure 6, A–C), suggesting that NRF2 signaling is critically involved in MICU1-mediated cold stress–induced ferroptosis. Meanwhile, a specific ferroptosis inhibitor (Fer-1) reduced cold stress–induced ferroptosis in WT LSECs (Figure 4, A, B, and D). These results indicate that MICU1-independent mechanisms can regulate ferroptosis in cold-stressed LSECs. Previous reports showed that iron affected cold-induced cell injury, especially in endothelial cells (38, 46), while adjunctive use of iron chelator in cold storage solution successfully protected microcirculation in rat small bowel grafts with prolonged cold ischemia (38). As LSECs are the predominant source of bone morphogenetic protein 6 for hepcidin and iron homeostasis (47, 48), the contribution of LSECs in iron metabolisms during cold storage is also worth investigating. Before ferroptosis was discovered, massive mitochondrial swelling was already reported in cold-stored human renal proximal tubular cells (34), while the involvement of mitochondria in ferroptosis is evident in calcium influx and iron accumulation (16, 49, 50). The discovery of ferroptosis should accelerate cold stress–induced transplant injury research, and traditional preservation solutions should be remade based on the recent progress. Adjunctive use of Fer-1 in UW solution can be readily applicable in clinical settings to protect endothelial cells during organ cold storage.

HMGB1, a major DAMP, which activates pattern recognition receptors, is instrumental in the liver IRI inflammatory cascade (2) and is passively released during various types of cell death, including ferroptosis (27). Indeed, Fer-1 treatment reduced HMGB1 release into the liver flush after cold storage (Figure 2C), while the decrease of HMGB1 release alleviated hepatic downstream inflammatory phenotype (IL-6, TNF-α, CXCL10) (Figure 2B). Furthermore, decreased inflammatory signature in cold-stored liver grafts reduced postreperfusion neutrophil and macrophage recruitment by suppressing the inflammatory chemokine program (Figure 1, A, B, and E). These results are consistent with the previous findings that ferroptosis enhanced DAMPs’ release, stimulating TLR4 signaling on graft endothelial cells to promote local neutrophil recruitment in cardiac grafts (22). Moreover, as HMGB1 release from liver cells can impact innate-adaptive immune crosstalk (2), protection of LSECs with Fer-1 treatment during liver cold storage in UW solution mitigated IRI-OLT by improving microcirculation and suppressing HMGB1 release.

A stress-inducible transcription factor, NRF2 is a major ferroptosis regulator while activating many cell protection genes and regulating oxidative defense, redox signaling, and iron homeostasis during ferroptosis (32, 51, 52). Although we have shown that NRF2 signaling impacted ROS-mediated LSEC insult (53), the role of NRF2 in cold-stressed LSECs remains unknown. In the current study, the disruption of NRF2 signaling increased MICU1 expression in LSECs, exacerbating cold stress–induced ferroptosis cell death pathway (Figure 4D). The NRF2/MICU1 axis might be mediated by ER calcium release, as deficient NRF2 signaling markedly enhanced ER stress response (Figure 4D). On the other hand, NRF2 may regulate MICU1 directly through miRNA miR181c (54, 55). Although further studies are needed to better appreciate the regulatory mechanisms, our findings revealed an essential contribution of NRF2 signaling in the MICU1-mediated ferroptosis pathway in cold-stressed LSECs. In addition to MICU1 levels, functional assessment of MICU1 should be also conducted in future studies.

We have validated that in addition to MICU1, NRF2 critically regulated a central player of ferroptosis, the antioxidative enzyme GPX4 (Figure 5, A and B). However, contrary to our expectation, cold stress did not affect the GCLC/GPX4 pathway in LSEC cultures (Supplemental Figure 4A), while in vivo treatment with GPX4 inhibitor, RSL3, failed to affect cold stress–induced liver damage (Figure 5, D−F). Although the GPX4 pathway was dispensable in the mechanism of cold stress–induced liver graft ferroptosis and LSEC damage, it was strongly upregulated in hepatocytes subjected to warm hypoxia/reoxygenation in our ongoing study (H Kojima, unpublished observations). Thus, distinct cell type–specific molecular mechanisms may contribute to ferroptosis cell death pathway in response to cold or warm IR stress in OLT recipients.

We have shown that MDA expression correlated positively with HMGB1 levels in the liver flush from discarded human livers (Figure 8C), consistent with the ability of HMGB1 to be released during ferroptosis (27). A recent study reported that plasma MDA was associated with the severity of multiorgan dysfunction and the probability of death in human patients (31). Thus, MDA/HMGB1 levels released from the damaged cells into the organ perfusate or detected in the recipient plasma may serve as surrogate markers of lipid peroxidation and ferroptosis in clinical practice.

Nonalcoholic fatty liver disease (NAFLD) affects LSEC phenotypes. The loss of fenestration, observed in cold-stored liver grafts by scanning electron microscopy (Figure 2H), can also be seen during early NAFLD development, i.e., at the steatosis stage in human livers (56). In addition, during the progression of steatohepatitis, LSECs become proinflammatory, characterized by the increase of cytokine/chemokine release and upregulation of adhesion molecules (56). As microcirculatory dysfunction represents the main mechanism of IRI-triggered hepatocellular damage in steatotic livers (10), protecting LSECs during cold storage may lead to the safe usage of steatotic livers. Considering worldwide donor organ shortage and the need to use “marginal” steatotic liver grafts, adjunct Fer-1 treatment to protect LSECs during cold storage might improve OLT outcomes and expand the organ pool available for lifesaving surgery. Toward its clinical application, the optimal concentration, time-dependent effects, and off-target effects of Fer-1 treatment during cold storage should be investigated in future studies. In parallel, developing new ferroptosis-targeting drugs is warranted. Indeed, recent studies explored compounds enhancing the stability and bioavailability of currently used ferroptosis inhibitors (57). With a promising report from phase I clinical trials with antiferroptosis agents in amyotrophic lateral sclerosis and Parkinson’s disease (58), improving donor organ quality by targeting ferroptosis cell death pathway during organ storage should be evaluated in parallel with refined organ preservation procedures.

In conclusion, we have documented the role of ferroptosis programmed cell death pathway in OLT outcomes in mice and humans. Inhibition of ferroptosis during ex vivo liver cold storage selectively protected LSECs from MICU1-driven cell death in an NRF2-dependent manner. Consistent with the regulatory function of NRF2 in ferroptosis during donor organ cold storage, enhanced liver NRF2 levels reduced lipid peroxidation, mitigated liver IRI, and lowered EAD incidence in human OLT recipients. This translational study provides the rationale for a clinically applicable therapeutic strategy using adjunct ferroptosis inhibitors during organ preservation to “rejuvenate” donor liver grafts by protecting LSECs from cold stress–induced hepatocellular damage.

## Methods

### Clinical liver transplant study.

We performed a retrospective analysis of 60 adult patients who underwent OLT (May 2013–August 2015) and received routine standard-of-care and immunosuppressive therapy. Recipients who underwent retransplantation were excluded. Donor livers, procured from donation after a brain or cardiac death, were stored in UW solution (Niaspan, Bristol Myers Squibb). Tru-Cut needle biopsies from the left liver lobe were obtained after cold storage at the back table (before implantation). Hepatic biopsies were screened by Western blots with β-actin normalization for NRF2, MICU1, and MDA expression. Cold ischemia time was defined as the time from the perfusion of the donor liver with UW solution to its removal from the cold storage for implantation. Warm ischemia time was defined as the time from removal from cold storage to establishing liver graft reperfusion. Recipient blood was collected before transplant and at POD 1–7. Liver injury was evaluated by serum AST and ALT levels. The incidence of EAD was defined as described (59).

Eight discarded human livers were obtained from OneLegacy Procurement Organization. Liver and flush samples obtained by perfusing those livers with UW solution (4 L) through a catheter inserted into the portal vein were analyzed with Western blots and immunostaining.

### Animals.

WT and NRF2-KO mice, purchased from The Jackson Laboratory, were on a C57BL/6 background and studied at 8–11 weeks of age. Animals were housed in the UCLA animal facility under pathogen-free conditions. They received humane care according to the criteria outlined in the NIH *Guide for the Care and Use of Laboratory Animals* (National Academies Press, 2011).

### Mouse OLT and liver cold storage.

We used a mouse model of liver cold storage followed by OLT, as described by our group (60). To mimic marginal human OLT, donor livers were stored in UW solution (4°C/18 h) and transplanted to syngeneic recipients to investigate the hepatocellular injury itself, without the confounding effects of allograft rejection response. Liver graft and plasma samples were collected 6 hours after reperfusion, the peak of hepatocellular damage in this model. Separate recipient groups were monitored for OLT survival. The sham group underwent the same procedures except for OLT. In separate experiments, donor livers were incubated (at indicated concentrations) with Fer-1 (SML0583, MilliporeSigma), RSL3 (SML2234, MilliporeSigma), or MCU-i4 (S9842, Selleck Chemicals, 10 μM) throughout 18 hours of cold storage. To investigate the impact of cold stress alone, some livers and liver flush (after infusion of 2 mL of PBS through a cuff placed at the portal vein) were obtained right after liver cold storage.

### Isolation and culture of hepatocytes and LSECs.

Primary mouse hepatocytes from WT or NRF2-KO donors, isolated by a 2-step collagenase perfusion method (61), were cultured with/without cold stress (4°C) for 3 hours or 6 hours, as described (37).

Primary mouse LSECs were isolated from WT or NRF2-KO and cultured, as described (62, 63). Briefly, isolated LSECs were cultured in EGM-2 BulletKit medium (Lonza) supplemented with 20 ng/mL mouse VEGF (R&D Systems, Bio-Techne). Cultured plates were washed after 4−6 hours’ adhesion to remove debris/dead cells. The isolated LSECs were cultured overnight in the presence of VEGF, and their purity was confirmed to be 85%–90% with immunofluorescence of stabilin-2 (Supplemental Figure 7). LSECs at day 1 were subjected to cold stress (4°C) for the indicated periods (3 hours or 6 hours). Fer-1 (SML0583, MilliporeSigma) and MCU-i4 (S9842, Selleck Chemicals) were used at 10 μM according to previous reports (43, 64). Dead hepatocytes and LSECs, detected with propidium iodide (1 μg/mL for 5 minutes), were assessed with a fluorescence microscope.

### Liver enzyme assays.

AST/ALT levels in plasma and liver flush were measured with AST/ALT Liquid Reagent Kit (Teco Diagnostics) and validated with Validate GC3 (Maine Standards Company). LDH levels in plasma and liver flush were measured with LDH assay kit (LifeSpan BioSciences) according to the manufacturer’s protocol.

### OLT histology and IRI grading.

Formalin-fixed, paraffin-embedded liver sections (4 μm) were stained with H&E. The severity of IRI was graded using Suzuki’s criteria (28).

### TUNEL assay.

Apoptotic/necrotic cells in liver sections (4 μm) were detected by In Situ Apoptosis Detection Kit (Takara Bio). Results were scored semiquantitatively by blindly counting the number of positive cells in 10 HPF/section (original magnification, ×400).

### Scanning electron microscopy.

Liver grafts cut into 5 mm^3^ pieces were fixed with 2% glutaraldehyde and 4% paraformaldehyde at 4°C overnight. After postfixation with 2% osmium tetroxide for 2 hours and gold-sputter coating, the specimens were examined with a scanning electron microscope (Nova NanoSEM 230, FEI).

### Western blot assay.

Proteins were extracted from tissue/cell samples, and their concentration was measured (BCA Protein Assay Kit, Thermo Fisher Scientific). An equal amount of protein was electrophoresed, blotted, incubated with primary antibodies and secondary HRP-conjugated antibodies, and developed. The relative value was normalized by β-actin. The following primary and secondary antibodies were used in this study: HMGB1 (6893/D3E5), CHOP (2895/L63F7), CBARA1/MICU1 (12524/D4P8Q), NRF2 (12721/D1Z9C), cleaved caspase-3 (9664/5A1E), RIP3 (95702/D4G2A), β-actin (4970/13E5) anti-rabbit IgG (HRP-linked antibody 7074), and anti-mouse IgG (HRP-linked antibody 7076) (all from Cell Signaling Technology); MDA (ab27642, Abcam); MDA (ab243066/11E3, Abcam); NRF2 (ab137550, Abcam); CD41/integrin αIIb (ab134131/EPR4330, Abcam); GCLC (ab190685/EP13475, Abcam); and GPX4 (MAB5457/565320, R&D Systems, Bio-Techne). To compare target protein expression in multiple human OLT samples, densitometry quantification was conducted, as reported (60). One of the biopsy samples expressing all target proteins was assigned as a control sample. An equal amount of protein lysate from each sample was applied to each well or gel, and the target band intensity was expressed as relative band intensity to that of the positive control in the same gel. The target relative protein value was further normalized according to β-actin intensity.

### qRT-PCR analysis.

RNA extracted with the RNeasy Mini Kit (QIAGEN) was reverse-transcribed into cDNA. Quantitative PCR was performed using QuantStudio 3 (Applied Biosystems, Thermo Fisher Scientific). The primer sequences are listed in Supplemental Table 3. The expression of the target gene was normalized to the housekeepers β-actin, HPRT, or GAPDH.

### Immunofluorescence/immunohistochemistry.

Mouse liver samples and isolated cells were stained with rabbit anti-CD68 Ab (ab12521, Abcam), rat anti-Ly6G (551459, BD Biosciences), rat anti–stabilin-2 (D317-3/34-2, Medical & Biological Laboratories), mouse anti-MDA (ab243066/11E3, Abcam), goat anti-albumin (A90-134A, Bethyl Laboratories), rat anti-CLEC4F (MA5-24113/370901, Invitrogen, Thermo Fisher Scientific), and rabbit anti-NRF2 (12721/D1Z9C, Cell Signaling Technology). For mouse-on-mouse staining, an unconjugated affinity-purified F(ab) fragment anti-mouse IgG (H+L) (ab6668, Abcam) was applied and incubated for 1 hour at room temperature to block endogenous IgG (65). Human liver samples were stained with rabbit anti-LYVE1 (ab10278, Abcam) and mouse anti-MDA (ab243066/11E3, Abcam). Signals were visualized with secondary Alexa Fluor antibodies: Alexa Fluor 488–conjugated donkey anti-mouse IgG (A-21202, Invitrogen, Thermo Fisher Scientific), Alexa Fluor 488–conjugated donkey anti-rabbit IgG (A-21206, Invitrogen, Thermo Fisher Scientific), Alexa Fluor 488–conjugated goat anti-rat IgG (A-11006, Invitrogen, Thermo Fisher Scientific), Alexa Fluor 555–conjugated donkey anti-rabbit IgG (A-31572, Invitrogen, Thermo Fisher Scientific), Alexa Fluor 555–conjugated goat anti-rat IgG (A-21434, Invitrogen, Thermo Fisher Scientific), and Alexa Fluor 555–conjugated goat anti-mouse IgG (A-21422, Invitrogen, Thermo Fisher Scientific). Integrin αIIb was stained in the mouse liver samples using rabbit anti-CD41 Ab (ab134131/EPR4330, Abcam) as the primary and HRP conjugated as the secondary (Dako Envision plus kit, K4065, Agilent Technologies).

### Statistics.

For mouse experiments, comparisons between 2 or more groups were assessed with Student’s *t* test or 1-way ANOVA followed by post hoc test (Tukey’s HSD test or Dunnett’s multiple comparisons test), respectively. Cumulative survival rates were evaluated using Kaplan-Meier methods, and the curves were compared using the log-rank test. For human data, continuous values were analyzed by the Mann-Whitney *U* test and categorical variables by Fisher’s exact test. Spearman’s correlation coefficient (*r*) was used to evaluate the strength of the linear relationship between variables. All *P* values were 2 tailed, and *P* less than 0.05 was considered statistically significant. GraphPad Prism 9 was used for statistical analyses.

### Study approval.

All human studies were approved by the UCLA Institutional Research Board (IRB protocol 13-000143 and 18-000216), and written informed consent was received from participants before inclusion in the study. All animal experiments were approved by the UCLA Animal Research Committee (ARC 1999-094).

### Data availability.

Original data are provided in the Supporting Data Values file.

## Author contributions

H Kojima and JWKW conceived and designed the study. H Kojima, HH, KK, TI, TT, SY, and KJD conducted experiments. H Kojima conducted mouse surgical procedures. H Kojima, HH, KK, TI, TT, SY, H Kitajima, TO, FMK, and DGF acquired data. H Kojima, HH, KK, TI, TT, SY, and KJD analyzed data. H Kojima and JWKW drafted the manuscript. JWKW obtained funding. All authors read and approved the manuscript.

## Supplementary Material

Supplemental data

Supporting data values

## Version 1 02/08/2024

Electronic publication
