# Supplementary material for: Cold stress–induced ferroptosis in liver sinusoidal endothelial cells determines liver transplant injury and outcomes
Source: JCI Insight. 2024 Feb 8;9(3):e174354. doi: 10.1172/jci.insight.174354 (PMC10967411; doi:10.1172/jci.insight.174354)
Supplement: Supplemental data [file jciinsight-9-174354-s131.pdf]

Supplemental material

Cold stress-induced ferroptosis in liver sinusoidal endothelial cells determines liver transplant injury and outcomes

Hidenobu Kojima[1]*, Hirofumi Hirao[1]*, Kentaro Kadono[1], Takahiro Ito[1], Siyuan Yao[1], Taylor Torgerson[1], Kenneth J. Dery[1], Hiroaki Kitajima[2], Takahiro Ogawa[2], Fady M. Kaldas[1], Douglas G. Farmer[1], and Jerzy W. Kupiec-Weglinski[1]

[1]The Dumont-UCLA Transplantation Center, Department of Surgery, Division of Liver and Pancreas Transplantation, David Geffen School of Medicine at UCLA, Los Angeles, CA 90095

[2]Weintraub Center for Reconstructive Biotechnology, Division of Regenerative and Reconstructive Sciences, UCLA School of Dentistry, Los Angeles, CA 90095

*These authors contributed equally to this work.

Table of contents

Supplemental Table 1……………………………………………………………………………………3

Supplemental Table 2A……………………………………………………………………………………4

Supplemental Table 2B……………………………………………………………………………………5

Supplemental Table 3……………………………………………………………………………………6

Supplemental Figure 1……………………………………………………………………………………7

Supplemental Figure 2……………………………………………………………………………………8

Supplemental Figure 3……………………………………………………………………………………9

Supplemental Figure 4……………………………………………………………………………………10

Supplemental Figure 5……………………………………………………………………………………11

Supplemental Figure 6……………………………………………………………………………………12

Supplemental Figure 7……………………………………………………………………………………13

Supplemental Table 1: Discarded human livers: Donor demographic data and organ information

Case	Age (years)	Gender	BMI (kg/m^2)	Race
1	51	Female	43.3	Hispanic
2	52	Male	32.1	Hispanic
3	57	Female	26.8	African American
4	61	Female	32.3	White
5	32	Male	20.4	Hispanic
6	60	Female	30.6	African American
7	46	Female	25.8	Hispanic
8	57	Female	21.2	White

Case	DBD or DCD	Reason for discard	DM	HTN	CAD	Cold storage time (minutes)
1	DBD	Steatosis	+	+	−	538
2	DBD	Unsuitable vessel condition	+	+	+	823
3	DBD	Unsuitable vessel condition	+	+	−	402
4	DBD	Steatosis	−	+	−	1105
5	DBD	Steatosis	−	−	−	495
6	DBD	Steatosis	−	+	−	737
7	DBD	Steatosis	+	+	+	960
8	DCD	Long non-beat time	−	+	−	384

BMI; body mass index, DBD; donor after brain death, DCD; donor after circulatory death, DM; diabetes mellitus, HTN; hypertension, CAD; coronary artery disease

Supplemental Table 2: Demographic data and clinical parameters of patients classified by low vs. high liver graft NRF2 expression

A. Liver graft recipients: Demographic data and clinical parameters

Variables	Low NRF2 (n=30)	High NRF2 (n=30)	*P* value
Age (years)	59 (29-73)	59 (33-73)	0.937
Sex (M/F)	19 (48.7%) / 11 (52.4%)	20 (51.3%)/ 10 (47.6%)	> 0.999
Race			0.721
White	15 (50.0%)	18 (60.0%)	
Hispanic	13 (43.3%)	9 (30.0%)	
Black	0 (0%)	0 (0%)	
Asian	1 (3.3%)	1 (3.3%)	
Others	1 (3.3%)	2 (6.7%)	
BMI (kg/m^2)	27.8 (17.9-40.0)	24.7 (14.5-47.5)	0.476
Disease etiology			0.532
HBV	1 (3.3%)	2 (6.7%)	
HCV	14 (46.7%)	11 (36.7%)	
Alcohol	2 (6.7%)	5 (16.7%)	
NASH	3 (10.0%)	4 (13.3%)	
Malignant tumor	2 (6.7%)	0 (0%)	
Others	8 (26.7%)	8 (26.7%)	
HCC (with/without)	14 (46.7%)/ 16 (53.3%)	11 (36.7%)/ 19 (63.3%)	0.601
ABO			0.237
Identical	27 (90.0%)	30 (100.0%)	
Compatibel	3 (10.0%)	0 (0%)	
MELD score	31 (9-40)	29 (14-44)	0.970
Pretransplant AST (IU/L)	74 (26-6967)	71.5 (23-1283)	0.608
Pretransplant ALT (IU/L)	51 (13-6168)	35 (11-617)	0.297
Pretransplant T-bil (mg/dL)	3.4 (0.3-59.1)	8.1 (0.5-49.2)	0.169
Pretransplant PT-INR	1.5 (1.0-2.9)	1.7 (1.1-3.2)	0.271

BMI; body mass index, HBV; hepatitis B virus, HCV; hepatitis C virus, NASH; nonalcoholic steatohepatitis, HCC; hepatocellular carcinoma

B. Liver graft donors: Demographic data and clinical parameters

Variables	Low NRF2 (n=30)	High NRF2 (n=30)	*P* value
Age (years)	47 (16-66)	42 (13-62)	0.582
Sex (M/F)	14 (46.7%)/ 16 (53.3%)	13 (43.3%)/ 17 (56.7%)	> 0.999
Race			0.512
White	17 (56.7%)	20 (66.7%)	
Hispanic	9 (30.0%)	8 (26.7%)	
Black	2 (6.7%)	0 (0%)	
Asian	2 (6.7%)	2 (6.7%)	
Others	0 (0%)	0 (0%)	
BMI (kg/m^2)	26.9 (19.7-38.4)	25.6 (13.4-42.6)	0.792
Pretransplant AST (IU/L)	35 (10-314)	34 (12-189)	0.179
Pretransplant ALT (IU/L)	28 (8-403)	23 (8-669)	0.144
Pretransplant T-bil (mg/dL)	0.8 (0.3-4.9)	0.6 (0.2-2.9)	0.054
Pretransplant PT-INR	1.3 (1.0-2.0)	1.2 (1.0-1.7)	0.619
Cold ischemic time (min)	426 (215-762)	432 (150-747)	0.945
Warm ischemic time (min)	55 (35-78)	49 (25-79)	0.653
DCD	2 (6.7%)	1 (3.3%)	> 0.999

BMI; body mass index, DCD; donor after circulatory death

Supplemental Table 3: Primer sequences for real-time reverse transcription PCR (mouse study)

Gene	Forward	Reverse
MCP1	5’-CATCCACGTGTTGGCTCA-3’	5’-GATCATCTTGCTGGTGAATGAGT-3’
CXCL1	5’-ACCCAAACCGAAGTCATAG-3’	5’-TTGTATAGTGTTGTCAGAAGC-3’
CXCL2	5’-ACTTCAAGAACATCCAGAG-3’	5’-CTTTCCAGGTCAGTTAGC-3’
CXCL10	5’-GCTGCCGTCATTTTCTGC-3’	5’-TCTCACTGGCCCGTCATC-3’
IL-6	5’-GTACCATAGCTACCTGGAGT-3’	5’-GGAAATTGGGGTAGGAAGGA-3’
TNF-α	5’-CCTATGTCTCAGCCTCTTCT-3’	5’-TTGGGAACTTCTCATCCCTT-3’
CHOP	5’-CTGCCTTTCACCTTGGAGAC-3’	5’-CGTTTCCTGGGGATGAGATA-3’
HIF-1α	5’-AGGAGCCTGATGCTCTCACTCT-3’	5’-TGTGTCATCGCTGCCAAAAT-3’
HRPT	5’-GATTAGCGATGATGAACCAGGTT-3’	5’-CCTCCCATCTCCTTCATGACA-3’

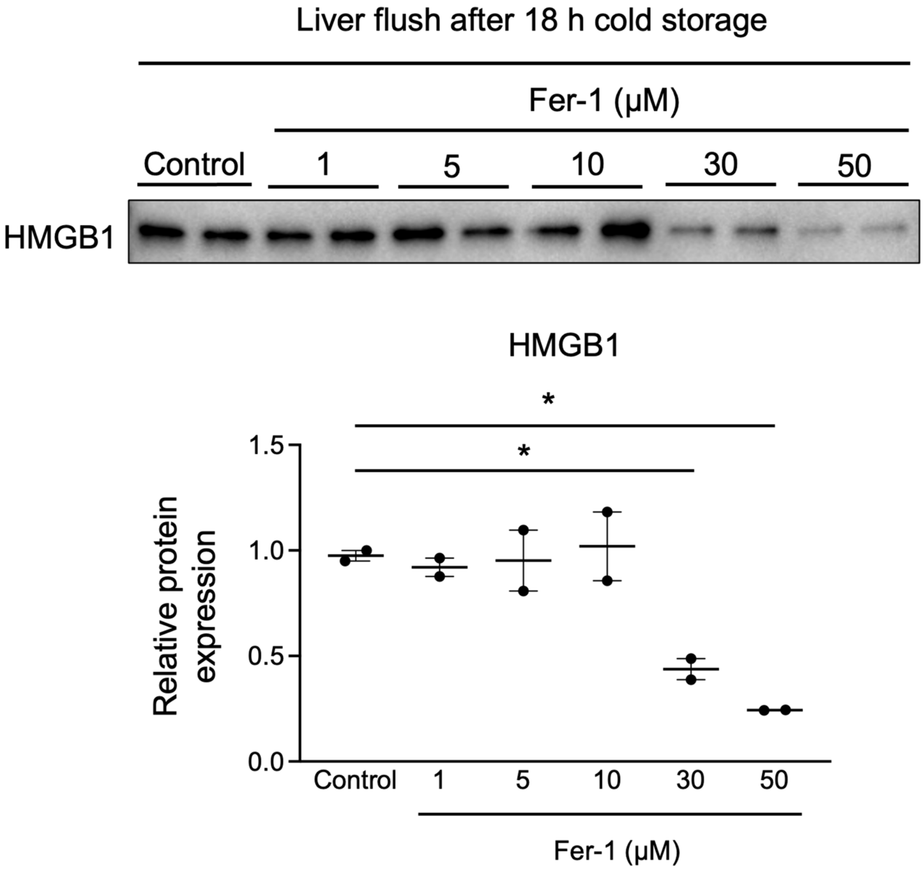

Supplemental Figure 1. Ferroptosis inhibitor (30μM) reduces HMGB1 levels in the liver flush. WT liver grafts stored in UW solution (4℃/18h) w/wo ferroptosis inhibitor (Ferostatin-1; Fer-1) were perfused with PBS (2mL) through a cuff placed at the portal vein to collect liver flush from inferior vena cava. Western blot-assisted detection of HMGB1 in the liver flush (5μL) from cold-stored liver grafts (n=2/group). Data are shown as mean±SEM. **P*<0.05, one-way ANOVA followed by Tukey's HSD test.

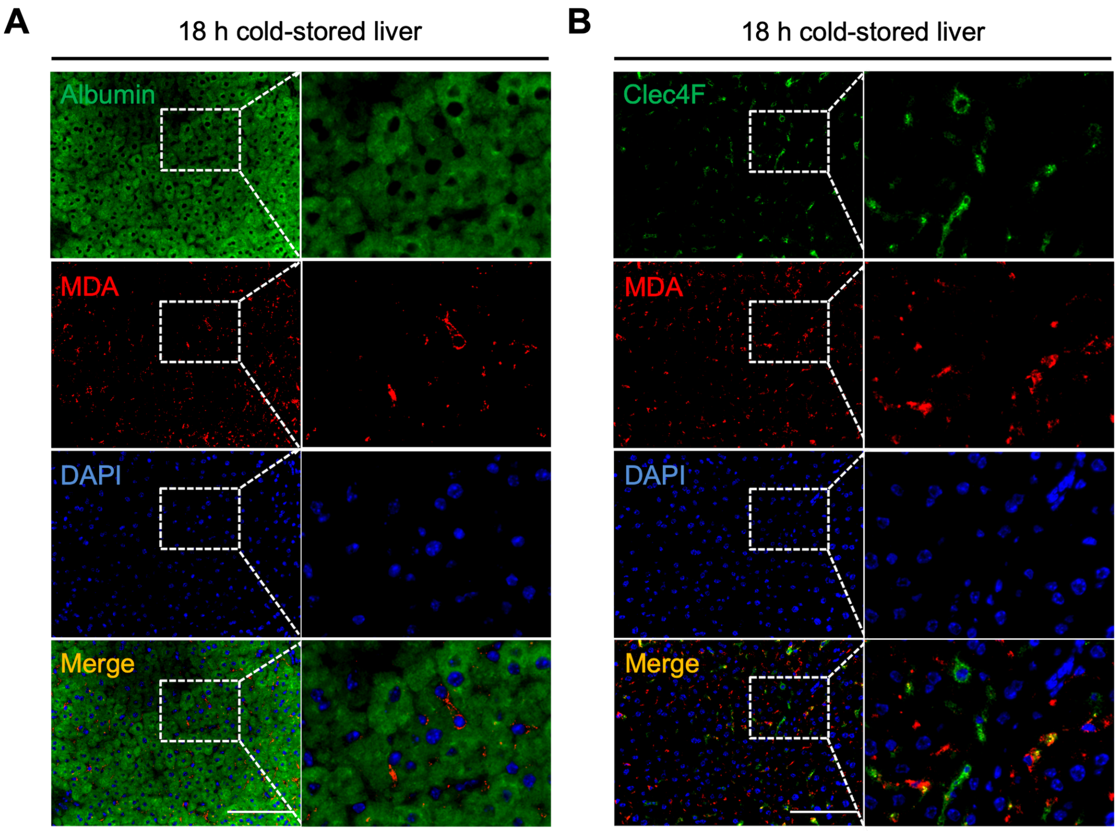

Supplemental Figure 2. Neither liver hepatocyte nor Kupffer cell represent the main source of MDA. (A, B) Representative (n=3/group) immunohistochemical staining of albumin/MDA (**A**) and Clec4F/MDA (**B**) in cold-stored mouse livers. Scale bars=100μm.

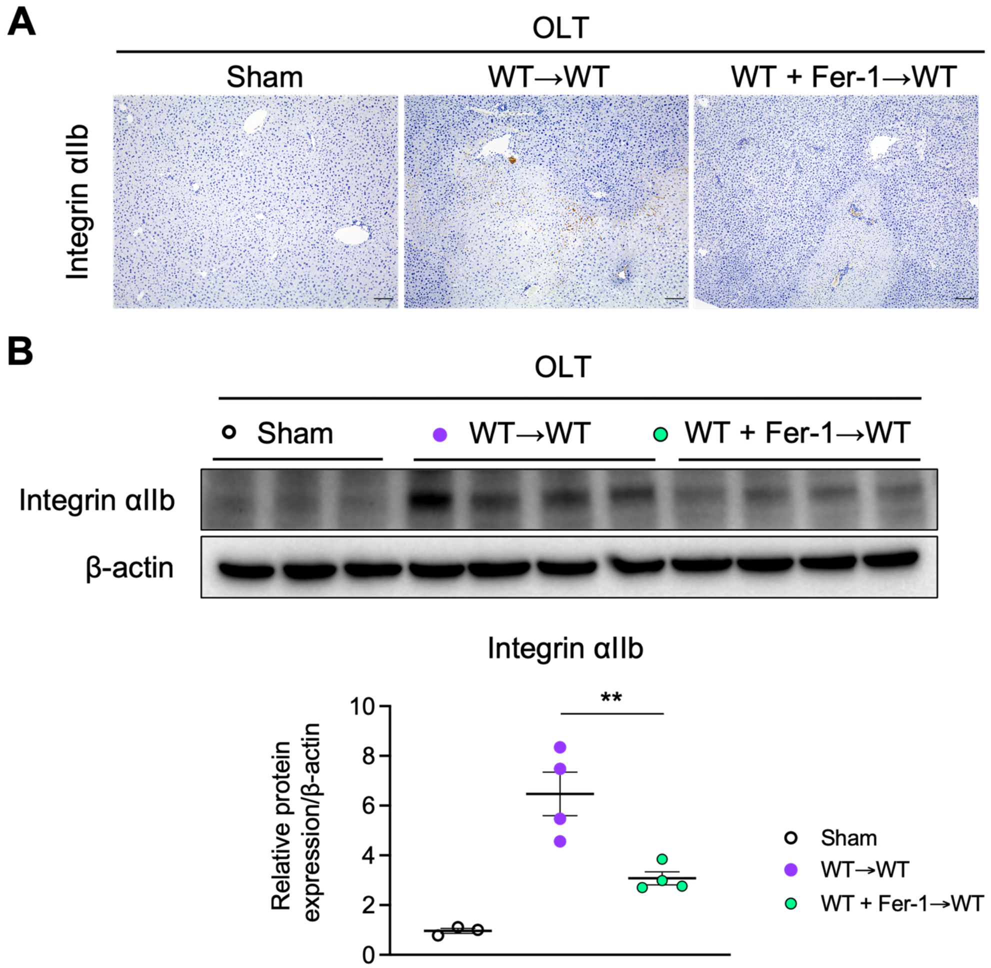

Supplemental Figure 3. Inhibition of ferroptosis during liver cold storage reduces platelet accumulation in mouse OLT. (A) Representative (n=5−6/group) integrin αIIb staining. Scale bars=100μm. **(B)** Western blot-assisted detection and relative intensity ratio of integrin αIIb in OLT. β-actin expression served as internal control and used for normalization (n=3−4/group). White circle: sham; purple circle: WT liver grafts; green circle: WT+Fer-1 liver grafts. Data are shown as mean±SEM. ***P*<0.01, Student's *t*-test.

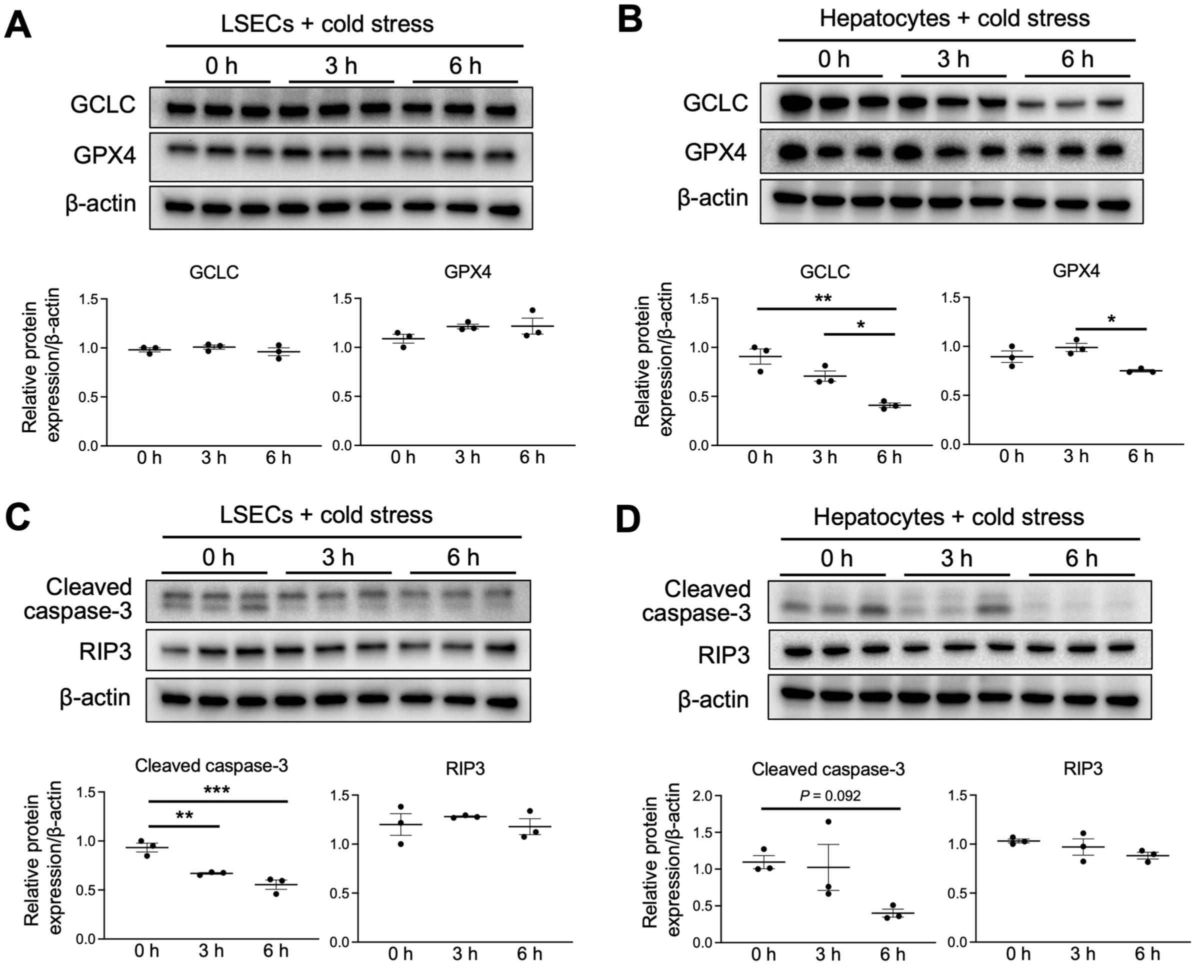

Supplemental Figure 4. Cold stress does not increase GCLC/GPX4, apoptosis, or necrosis in LSECs and hepatocytes. Western blot-assisted detection and relative intensity ratio of GCLC and GPX4 in **(A)** LSECs and **(B)** hepatocytes; cleaved caspase-3 and RIP3 in **(C)** LSECs and **(D)** hepatocytes after 6 hours of cold stress (4℃). β-actin expression served as internal control and used for normalization (n=3/group). Data are shown as mean±SEM. *P<0.05, **P<0.01, ***P<0.001, one-way ANOVA followed by Tukey's HSD test.

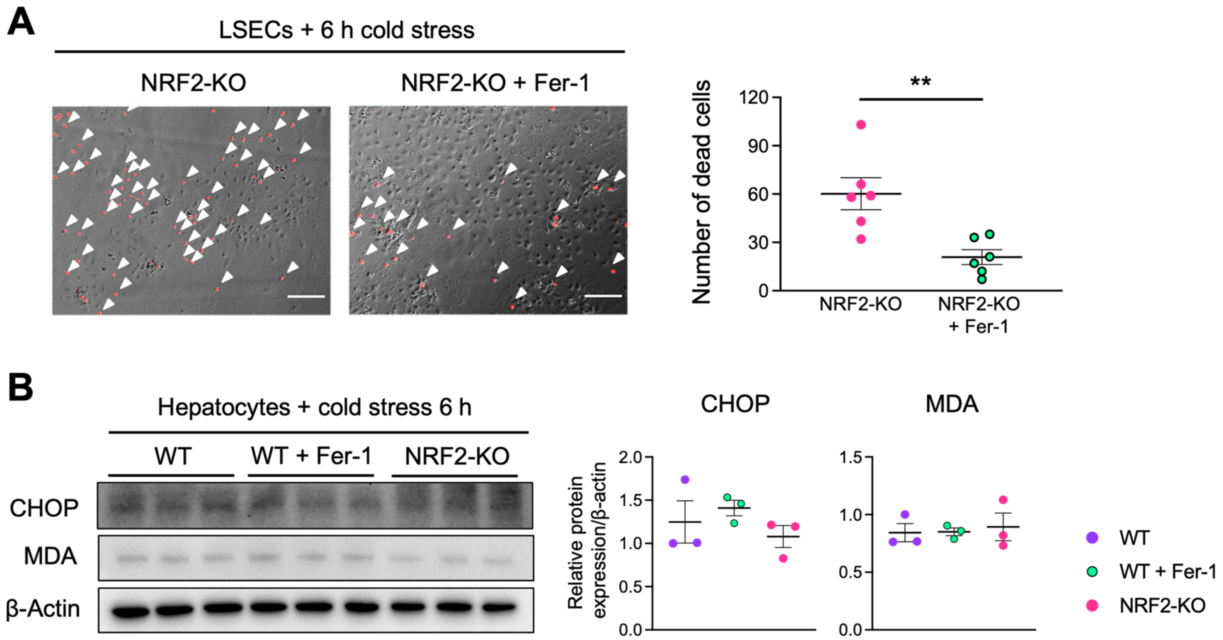

Supplemental Figure 5. NRF2 deficiency does not enhance ER stress or lipid peroxidation in hepatocytes. (A) Representative (n=6/group) images and quantification of dead cells in NRF2-deficient (NRF2-KO) LSECs w/wo ferroptosis inhibitor (Ferrostatin-1; Fer-1) after 6h cold stress (4℃). Arrowheads indicate dead cells. Scale bars=100 µm. **(B)** Western blot-assisted detection and relative intensity ratio of CHOP and MDA in hepatocyte culture after 6h of cold stress. β-actin expression served as internal control and used for normalization (n=3/group). Data are shown as mean±SEM. ***P* < 0.01, Student's *t*-test.

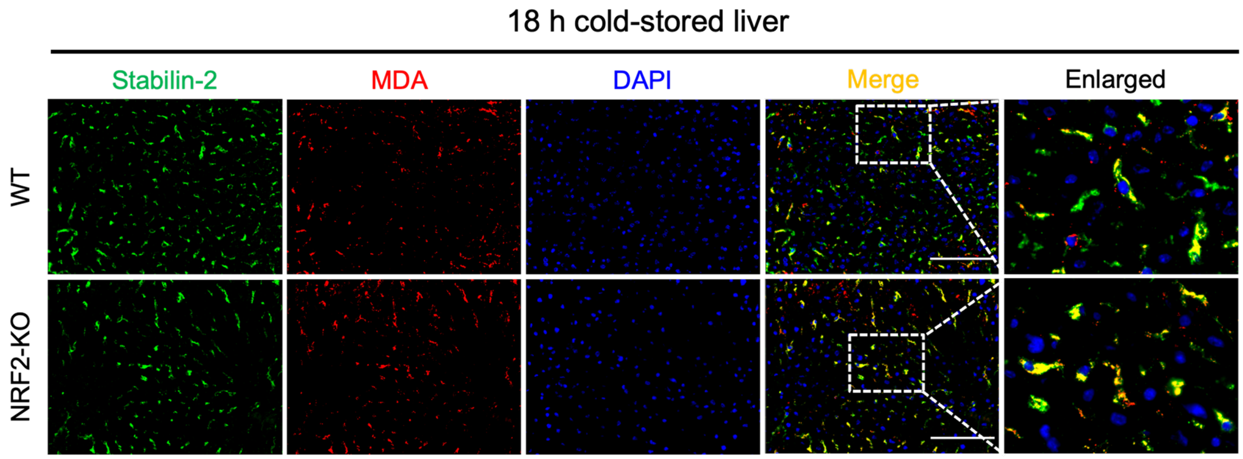

Supplemental Figure 6. MDA expression in LSECs of NRF2-deficient and WT liver grafts. Representative (n=3/group) immunohistochemical staining of stabilin-2/MDA in WT and NRF2-KO in cold-stored liver grafts (18h/4℃).

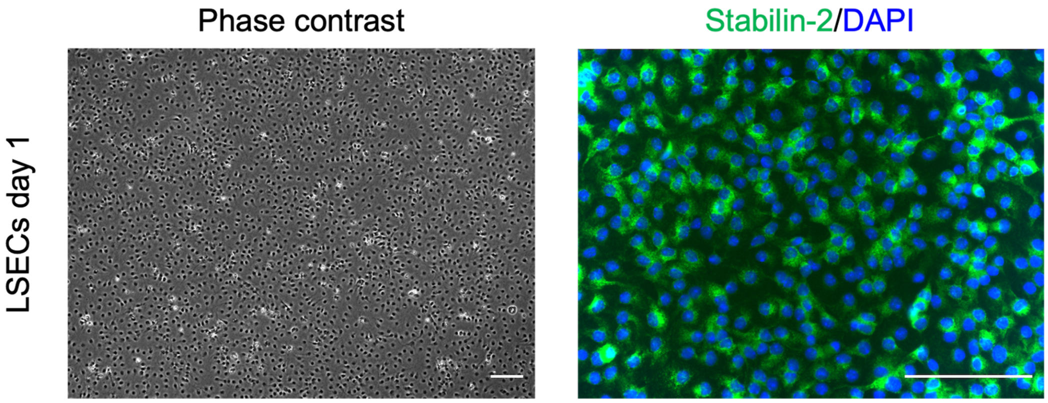

Supplemental Figure 7. The purity of isolated and cultured murine LSECs (>85−90%). Phase contrast image and immunohistochemical staining of stabilin-2 in LSECs at day 1 after isolation. Scale bars=100μm.
